# p90RSK-MAGI1 Module Controls Endothelial Permeability by Post-translational Modifications of MAGI1 and Hippo Pathway

**DOI:** 10.3389/fcvm.2020.542485

**Published:** 2020-11-13

**Authors:** Rei J. Abe, Hannah Savage, Masaki Imanishi, Priyanka Banerjee, Sivareddy Kotla, Jesus Paez-Mayorga, Jack Taunton, Keigi Fujiwara, Jong Hak Won, Syed Wamique Yusuf, Nicolas L. Palaskas, Jose Banchs, Steven H. Lin, Keri L. Schadler, Jun-ichi Abe, Nhat-Tu Le

**Affiliations:** ^1^Department of Cardiovascular Sciences, Center for Cardiovascular Regeneration, Houston Methodist Research Institute, Houston, TX, United States; ^2^Department of Pediatric Research, The University of Texas MD Anderson Cancer Center, Houston, TX, United States; ^3^Department of Cardiology, The University of Texas MD Anderson Cancer Center, Houston, TX, United States; ^4^Department of Cellular and Molecular Pharmacology, University of California, San Francisco, San Francisco, CA, United States; ^5^Department of Radiation Oncology, The University of Texas MD Anderson Cancer Center, Houston, TX, United States

**Keywords:** p90RSK, SUMOylation, Hippo pathway, EC permeability, MAGI1

## Abstract

Previously, we reported that post-translational modifications (PTMs) of MAGI1, including S741 phosphorylation and K931 de-SUMOylation, both of which are regulated by p90RSK activation, lead to endothelial cell (EC) activation. However, roles for p90RSK and MAGI1-PTMs in regulating EC permeability remain unclear despite MAGI1 being a junctional molecule. Here, we show that thrombin (Thb)-induced EC permeability, detected by the electric cell-substrate impedance sensing (ECIS) based system, was decreased by overexpression of dominant negative p90RSK or a MAGI1-S741A phosphorylation mutant, but was accelerated by overexpression of p90RSK, siRNA-mediated knockdown of *magi1*, or the MAGI1-K931R SUMOylation mutant. MAGI1 depletion also increased the mRNA and protein expression of the large tumor suppressor kinases 1 and 2 (LATS1/2), which inhibited YAP/TAZ activity and increased EC permeability. Because the endothelial barrier is a critical mediator of tumor hypoxia, we also evaluated the role of p90RSK activation in tumor vessel leakiness by using a relatively low dose of the p90RSK specific inhibitor, FMK-MEA. FMK-MEA significantly inhibited tumor vessel leakiness at a dose that does not affect morphology and growth of tumor vessels *in vivo*. These results provide novel insights into crucial roles for p90RSK-mediated MAGI1 PTMs and the Hippo pathway in EC permeability, as well as p90RSK activation in tumor vessel leakiness.

## Introduction

Endothelial cell-cell junctions are highly dynamic structures that regulate EC monolayer integrity and barrier function. A number of signaling pathways that regulate cell-cell junctions such as β-catenin phosphorylation and VE-cadherin degradation have been delineated (1–6). Rap1, a member of the Ras-like small GTPase family, has been recognized as a key regulator of cell-cell junctional formation at different levels through cadherins (7, 8). Rap1 also plays a role in the maintenance of cell-cell adhesion. Endothelial Rap1 activation inhibits EC permeability (9–12). In ECs, the formation of cell-cell contacts induced by Rap1 activation is hampered by the depletion of Membrane Associated Guanylate Kinase, WW and PDZ domain-containing protein 1 (MAGI1) (13). These observations suggest that MAGI1 is involved in the regulation of EC permeability.

MAGI1, a membrane-associated guanylate kinase (MAGUK) protein with an inverted domain structure 1, is expressed at sites of the cell-cell contact and associated with both tight and adherens junctions (13–15). MAGI1 functions as a scaffold protein (16) that regulates cell-cell adhesion and tight junction assembly through scaffolding trans-membrane proteins with the cytoskeleton (17). MAGI1 is composed of six PSD95/DiscLarge/ZO-1 (PDZ) domains, a guanylate kinase domain, and two WW (rsp5) domains flanked by the first and second PDZ domains (15). Through the PDZ domains, MAGI1 interacts with intracellular molecules such as phosphatase and tensin homolog deleted on chromosome 10 (PTEN) (18), Rho family nucleotide exchange factor (mNET1) (19), RapGEF2, and thyroid receptor interacting protein-6 (TRIP6) (13, 14, 18), to regulate multiple cellular functions and to stabilize the cell-cell junction. For example, the MAGI1 PDZ5 domain interacts with β-catenin and localizes to the cell-cell contact, where MAGI1 associates with a guanine nucleotide exchange factor (GEF) for Rap1 (PDZ-GEF1) and thus activating Rap1. MAGI1 WW domains can also bind a wide range of proteins, suggesting that MAGI1 serves as a platform for protein-protein interaction (20).

The core of the Hippo pathway in mammals is composed of the Ste20-like kinases (MST1/2), the large tumor suppressor kinases 1 and 2 (LATS1/2), the Yes-associated protein (YAP) and the transcriptional coactivator with PDZ-binding motif tafazzin (TAZ). YAP, known for its function as a transcription coactivator and oncoprotein, is located in the cytoplasm when phosphorylated, resulting in the inhibition of its transcriptional activity and effectively inactivating the protein (21). Through WW domain-mediated interactions, MST1/2 and LATS1/2 are phosphorylated (22), which inhibits the downstream YAP/TAZ transcription coactivators by inducing YAP phosphorylation and export from the nucleus to the cytoplasm (21, 23, 24). Mo et al. reported that the stimulation of protease-activated receptors (PARs) can activate YAP/TAZ by decreasing phosphorylation and increasing nuclear localization, and that PAR1 acts through G_12/13_ and Rho GTPase to inhibit the LATS1/2 kinase. These observations established the role of thrombin as a physiological signal for the Hippo pathway and implicated the Hippo-YAP as a key downstream signaling branch of PAR activation. However, it has also been reported that the WW domains on YAP bind the PPXY motifs on LATS1/2. Further, using affinity purification mass spectrometry and the proximity-dependent biotin identification (BioID) technique to systematically characterize protein interactions with the core components of the Hippo pathway, Couzens and Weiss et al. found that MAGI1 is one of the binding partners of LATS1, although the functional role of this binding has not been explored (25). Recent studies have shown that disturbed-flow, an atherogenic stimulus, induces nuclear translocation of YAP/TAZ leading to EC inflammation (26). Furthermore, it has been shown that YAP/TAZ is involved in EC permeability through regulation of VE-cadherin mediated BMP signaling (27, 28). However, to our best knowledge, the role of LATS1/2 in EC permeability has not been tested, and the relationship between MAGI1 and the Hippo pathway in regulating EC barrier function remains largely unknown. As a result, we also evaluated the role of MAGI1-LATS1/2-YAP signaling in regulating EC permeability in this study.

The p90 ribosomal S6 kinase (p90RSK) signaling cascade has been demonstrated to play an essential role in multiple cellular functions with the ability to phosphorylate and regulate the activity of many transcriptional factors and kinases. In a previous study, we found that p90RSK directly phosphorylates MAGI1 S741 and causes MAGI1 de-SUMOylation at K931, leading to EC activation (29). In this study, we will examine whether these p90RSK-dependent MAGI1 post-translational modifications (PTMs) impact EC barrier functions. We will also determine the relationship between the p90RSK-MAGI1 interaction and the Hippo pathway, and how the p90RSK-MAGI1 module controls EC barrier function.

Disruption of EC barrier integrity induces EC monolayer permeability and thus vascular leakage, which significantly lowers blood perfusion in tumors (30). Hyper-permeable tumor endothelium has been associated with tumor cell hypoxia, which is associated with chemotherapy and radiation resistance, as well as increased metastatic potential (31, 32). While tumor vascular permeability is known to be a critical mediator of multiple facets of tumor growth and cancer therapy, the contribution of p90RSK in regulating tumor EC permeability remains unclear. As such, we will also investigate the role of p90RSK activation in tumor vessel hyper-permeability *in vivo*.

## Results

### EC Monolayer Permeability Is Regulated by p90RSK Activation and MAGI1

**Figure 1:**
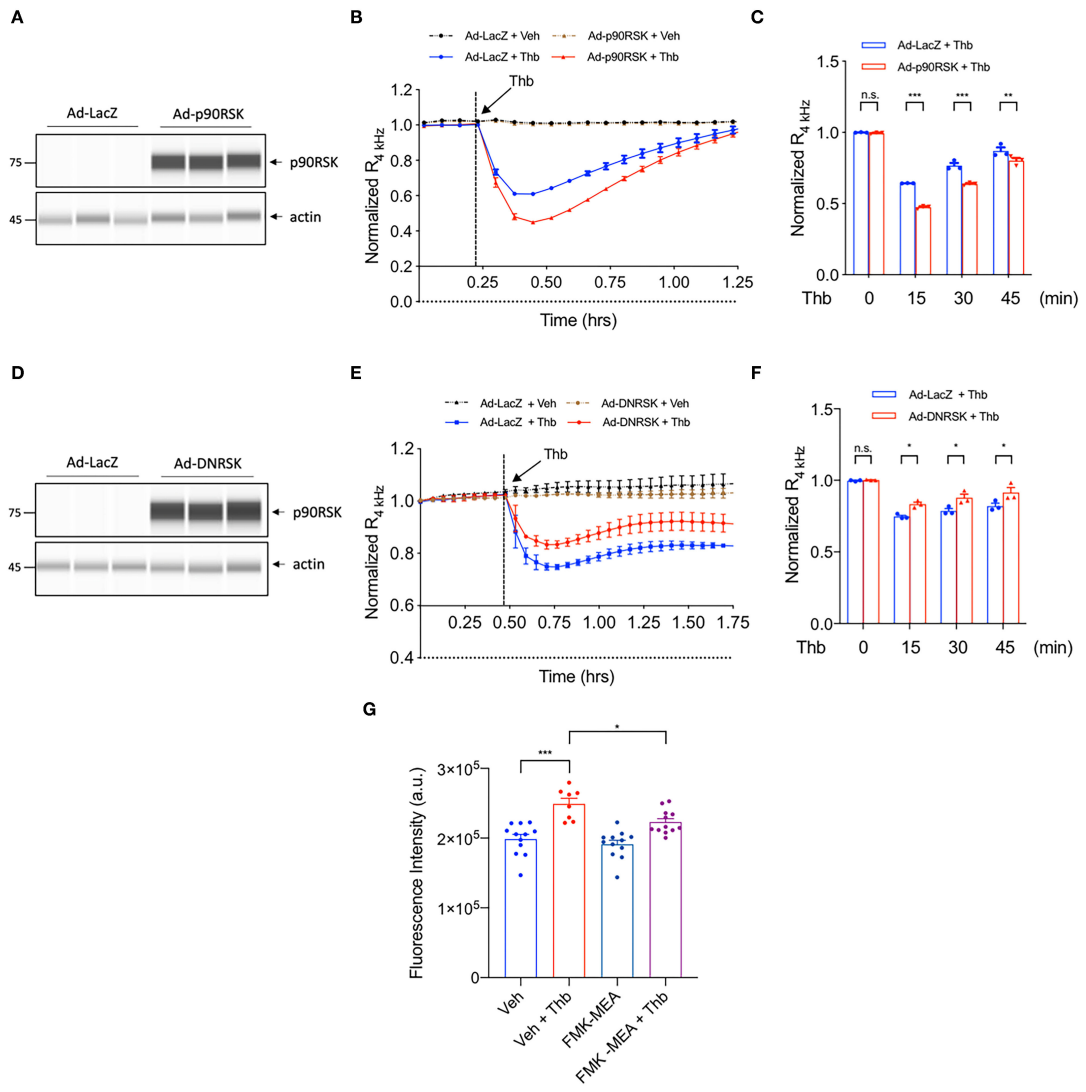
p90RSK increases EC permeability. **(A)** Automated capillary electrophoresis western analysis (WES analysis) of p90RSK protein expression level in lysates collected from human umbilical vein ECs (HUVECs) transduced with Ad-p90RSK or Ad-LacZ, β-actin served as a loading control. Protein bands are shown as pseudoblots. **(B)** Thb (10 U/mL)-mediated reduction in TEER values observed in the Ad-LacZ-transduced cells (blue) was accelerated in the Ad-p90RSK-transduced cells (red), as assessed by the ECIS system and shown as normalized resistance measured approximately every 4 min for indicated times. The dashed line indicates addition of Thb. **(C)** Graph demonstrates normalized resistance after Thb treatment at indicated times, relative to basal level (mean ± SEM, *n* = 3) **(D)** WES analysis of DNRSK protein expression level in lysates collected from HUVECs transduced with Ad-DNRSK or Ad-LacZ, β-actin was served as a loading control. Protein bands are shown as pseudoblots. **(E)** Thb (10 U/mL)-mediated reduction of TEER values observed in the Ad-LacZ-transduced cells (red) was inhibited in the Ad-DN-p90RSK-transduced cells (blue), as assessed by the ECIS system and shown as normalized resistance measured approximately every 4 min for indicated times. The dashed line indicates addition of Thb. A reduction in TEER indicates an increase in cell barrier permeability (33) through paracellular mechanism (34). **(F)** Graph demonstrates normalized resistance after Thb treatment at indicated times, relative to basal level (mean ± SEM, *n* = 3). Statistical significance was assessed using ANOVA followed by Bonferroni *post-hoc* testing for multiple group comparison. ***P < 0.001, **P < 0.01, and *P < 0.05. **(G)** Graph demonstrates that Thb (10 U/mL)-induced permeability in HUVECs, represented by fluorescence intensity measured in arbitrary units (a.u.), is inhibited by treatment with the specific p90RSK inhibitor FMK-MEA (10 uM). An increase in fluorescence intensity indicates an increase in cell barrier permeability (35, 36). Statistical significance was assessed using ANOVA followed by Bonferroni *post-hoc* testing for multiple group comparison. **P < 0.01.

To study a potential role for the p90RSK-MAGI1 signaling in EC barrier function, we used the electric cell-substrate impedance sensing (ECIS) based system to non-invasively measure trans-endothelial electrical resistance (TEER). Through this system, a decrease in TEER value indicates an increase in cell barrier permeability (33). We transduced adenovirus expressing p90RSK (Ad-p90RSK) or Lac Z (Ad-LacZ, control) to ECs, then stimulated the cells with Thb and measured TEER values. By comparison, we found that p90RSK overexpression significantly increases Thb-induced EC permeability as seen by the decreased TEER values in comparison to the control (Figures 1A–C). However, in ECs transduced with adenovirus expressing dominant negative p90RSK (Ad-DNRSK), the Thb-induced increase of EC permeability was inhibited, as shown by the higher TEER values in comparison to the control (Figures 1D–F) (33, 34). Through the FITC-Dextran trans-endothelial permeability assay, we also observed that Thb-induced EC permeability was decreased in ECs pretreated with FMK-MEA, a p90RSK specific inhibitor (Figure 1G). In this assay, this decrease in permeability is demonstrated by the inhibition of fluorescence seen in comparison to the Thb-treated control cells. Of note, we chose FMK-MEA because this compound is a potent, highly specific and irreversible inhibitor of p90RSK that covalently modifies the C-terminus kinase domain of p90RSK1, RSK2, and RSK4; as a result, it is more selective than other known p90RSK inhibitors (37). Cumulatively, this data suggests that p90RSK activation regulates EC permeability *in vitro*.

**Figure 2:**
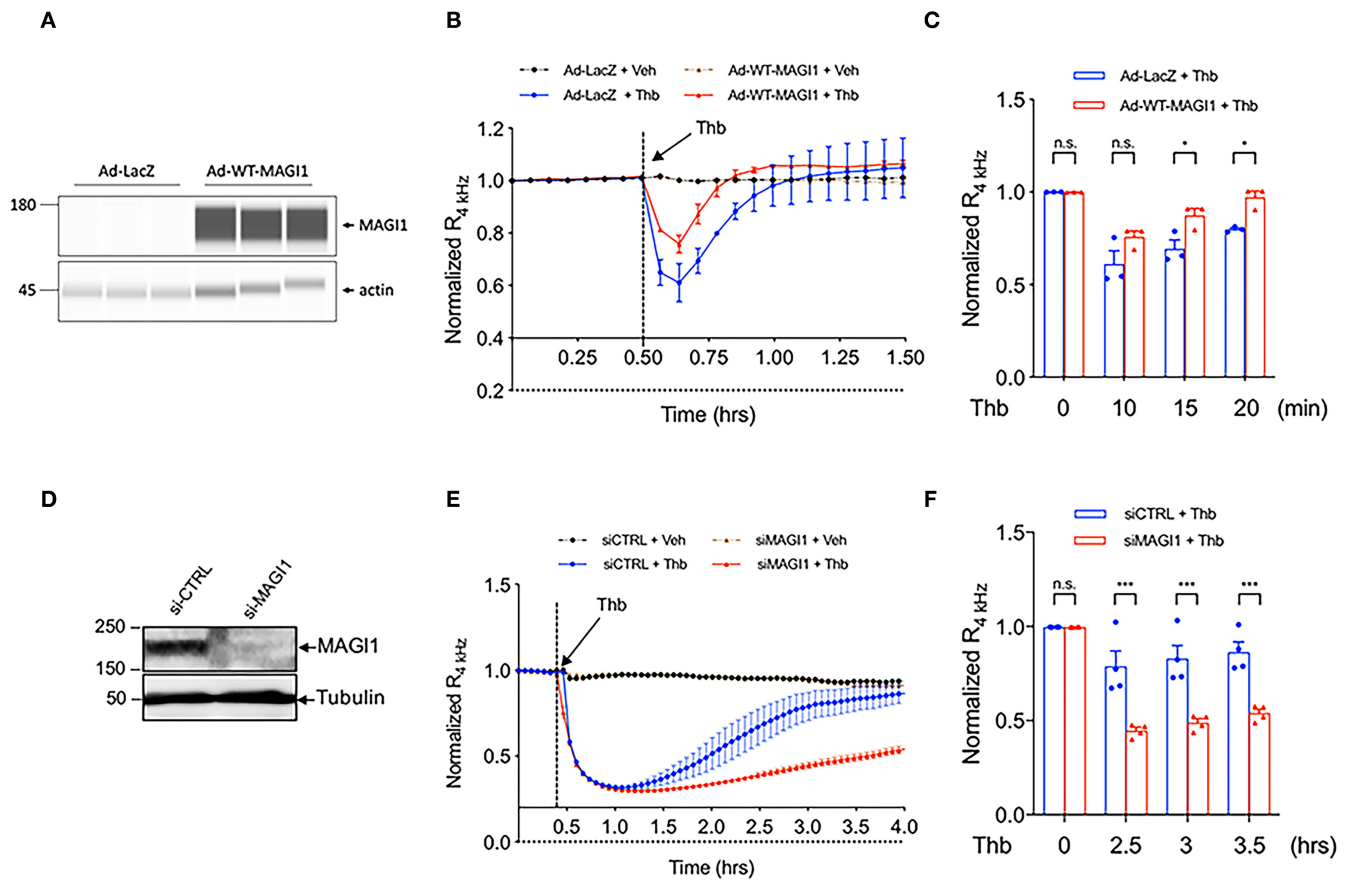
MAGI1 is required to maintain EC barrier function. **(A)** WES analysis of MAGI1 protein expression level in lysates collected from HUVECs transduced with Ad-MAGI1 or Ad-LacZ, β-actin served as a loading control. Protein bands are shown as pseudoblots. **(B)** Thb (10 U/mL)-mediated reduction in TEER values observed in cells transduced with Ad-LacZ (red) was inhibited in cells transduced with Ad-MAGI1 (blue), as assessed by ECIS system and shown as normalized resistance measured approximately every 4 min for indicated times. The dashed line indicates addition of Thb. **(C)** Graph demonstrates normalized resistance after Thb treatment at indicated times, relative to basal level (mean ± SEM, *n* = 3). **(D)** IB analysis of MAGI1 protein expression level in lysates collected from human aortic ECs (HAECs) treated with MAGI1 siRNA (siMAGI1) (100 nM, 48 h) or control siRNA (siCTRL), tubulin served as a loading control. **(E)** Thb (5 U/mL)-mediated reduction in TEER values observed in cells treated with siCTRL (blue) was increased to a greater extent in cells treated with siMAGI1 (red), as assessed by the ECIS system and shown as normalized resistance measured approximately every 4 min for indicated times. The dashed line indicates addition of Thb. A reduction in TEER values indicates an increase in cell barrier permeability (33) through paracellular mechanisms (34). **(F)** Graph demonstrates normalized resistance after Thb treatment at indicated times, relative to basal level (mean ± SEM, *n* = 4). Statistical significance was assessed using ANOVA followed by Bonferroni *post-hoc* testing for multiple group comparison. ***P < 0.001 and *P < 0.05.

Although the role of MAGI1 in barrier function was suggested by its interaction with JAM4 (17), whether MAGI1 regulates EC permeability was not previously confirmed. We transduced ECs with adenovirus expressing MAGI1 wild type (Ad-MAGI1-WT) or Ad-LacZ and found that Thb-induced EC permeability is decreased by MAGI1 overexpression (Figures 2A–C). In contrast, the depletion of MAGI1 induced by small interfering RNA (siRNA) significantly increases EC permeability (Figures 2D–F). This data indicates the critical role of MAGI1 in regulating EC barrier function.

### Both S741 Phosphorylation and K931 SUMOylation of MAGI1 Regulate EC Barrier Function

**Figure 3:**
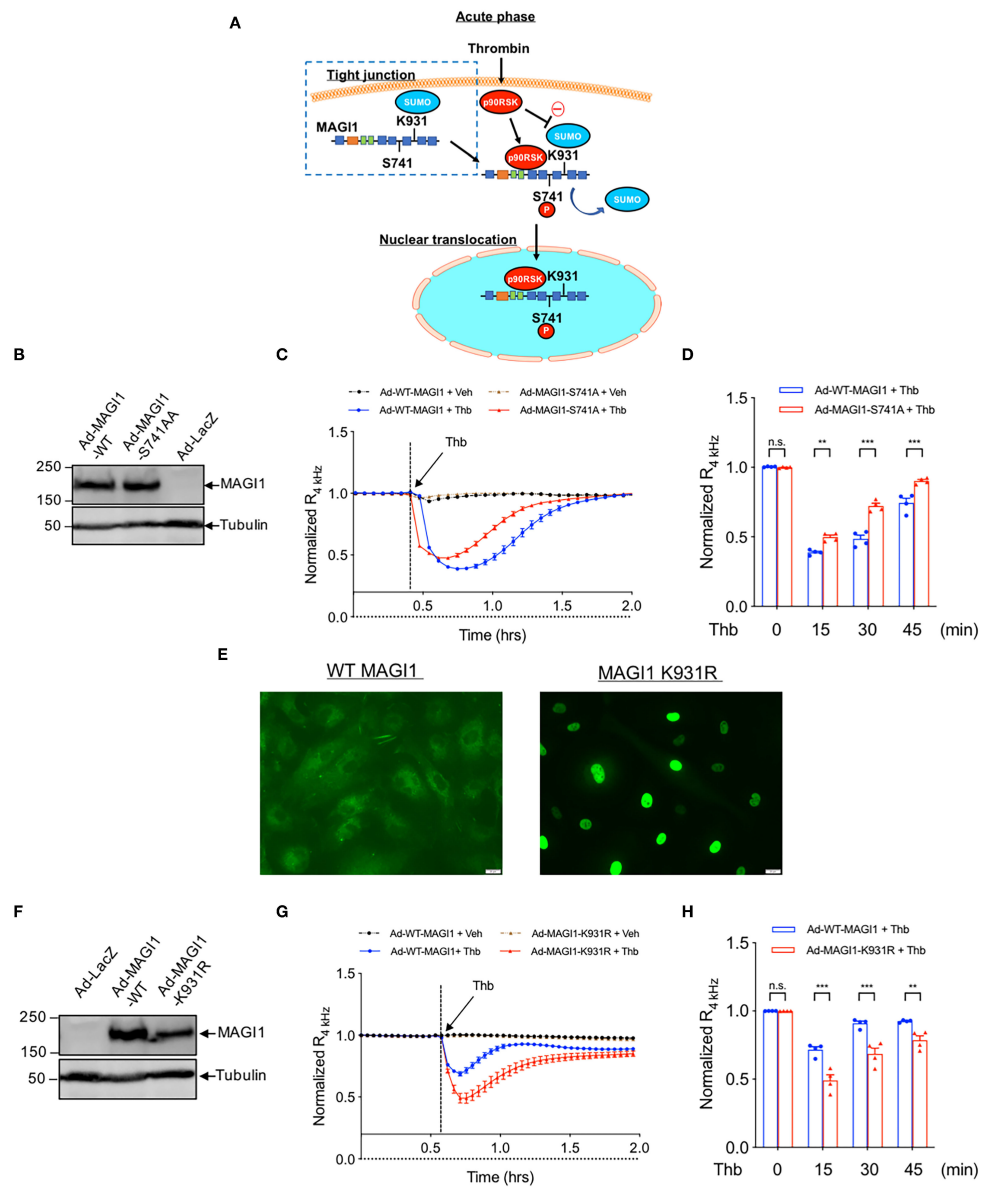
MAGI1 S741 phosphorylation and de-SUMOylation regulates EC permeability. **(A)** Schematic illustration of MAGI1 PTMs and cellular localization. **(B)** IB analysis of MAGI1 protein expression level in lysates collected from HAECs transduced with Ad-LacZ, -MAGI1-WT, or -S741A phosphorylation mutant, tubulin served as a loading control. **(C)** Thb (2.5 U/mL)-mediated reduction in TEER values observed in cells transduced with MAGI1-WT (blue) was significantly inhibited in cells transduced with the MAGI1-S741A phosphorylation mutant (red) as assessed by the ECIS system and shown as normalized resistance measured approximately every 4 min for indicated times. The dashed line indicates addition of Thb. **(D)** Graph demonstrates normalized resistance after Thb treatment at indicated times, relative to basal level (mean ± SEM, *n* = 4) **(E)** HUVECs transduced with Ad-Flag-MAGI1-WT or -K931R were treated with Thb (1 h) and then immunostained with a Flag antibody (green) **(F)** IB analysis of MAGI1 protein expression level in lysates collected from HAECs transduced by Ad-MAGI1 K931R mutant, Ad-MAGI1-WT, and Ad-LacZ, tubulin served as a loading control. **(G)** Thb (2.5 U/mL)-mediated reduction in TEER values observed in cells transduced with Ad-MAGI1 WT (blue) was increased to a greater extent in cells transduced with Ad-MAGI1 K931R mutant (red), as assessed by ECIS system and shown as normalized resistance measured approximately every 4 min for indicated times. The dashed line indicates addition of Thb. A reduction in TEER indicates an increase in cell barrier permeability (33) through paracellular mechanisms (34). **(H)** Graph demonstrates normalized resistance after Thb treatment at indicated times, relative to basal level (mean ± SEM, *n* = 4). Statistical significance was assessed using ANOVA followed by Bonferroni *post-hoc* testing for multiple group comparison. ***P < 0.001 and **P < 0.01.

We previously reported that MAGI1 is associated with p90RSK and that Thb induces MAGI1 S741 phosphorylation *via* p90RSK activation (29) (Figure 3A). To determine whether MAGI1 S741 phosphorylation is required for the Thb-induced increase in EC permeability, ECs transduced with either Ad-MAGI1-WT or -S741A mutant were stimulated with Thb and TEER values were measured (Figures 3B–D). We confirmed equal expression levels of MAGI1 in Ad-MAGI1-WT and -S741A transduced ECs (Figure 3B) and found a greater reduction of TEER values in the Ad-MAGI1-WT transduced group in comparison to the Ad-MAGI1-S741A mutant group (Figures 3C,D). Because a decrease in TEER values indicates an increase in EC permeability (33), this data demonstrates that EC permeability is significantly higher in the Ad-MAGI1-WT transduced ECs (Figure 3C, blue line) compared to that of the Ad-MAGI1-S741A transduced ECs (Figure 3C, red line). As such, the time required for basal-to-peak reduction is greater in the Ad-MAGI1-WT transduced ECs (blue line) than that of the Ad-MAGI1-S741A transduced ECs (red line). By comparison, Ad-MAGI1-WT transduced ECs may show a delayed response to Thb because the Ad-MAGI1 S741A transduced group eliminates the effects caused by MAGI1 S741 phosphorylation on EC permeability. However, this delay does not reach statistical significance. Overall, our data suggests that MAGI1 S741 phosphorylation promotes not only Rap1 and NF-kB activation (29) but also Thb-induced EC permeability.

In the same previous study, we also found that MAGI1 is SUMOylated at K931, and that disturbed flow and Thb significantly inhibits MAGI1 SUMOylation via promoting p90RSK-mediated SENP2 T368 phosphorylation (29), thus causing MAGI1 nuclear translocation (Figures 3A,E) (38). To examine whether MAGI SUMOylation and nuclear translocation regulates EC barrier function, we transduced ECs with Ad-MAGI1-WT or -K931R SUMOylation deficient mutant then stimulated the transduced ECs with Thb and measured TEER values. We confirmed equal expression levels of MAGI1 in both the Ad-MAGI1-WT and -K931R transduced ECs (Figure 3F) and found that Thb-induced EC permeability was increased to a greater extent by the Ad-MAGI1-K931R transduction (Figures 3G,H). Together with previous observations (29), these results indicate that MAGI1 K931 SUMOylation not only prevents MAGI1 nuclear translocation but also stabilizes barrier function.

### The Depletion of MAGI1 Up-Regulates LATS Expression and Inhibits YAP Expression

**Figure 4:**
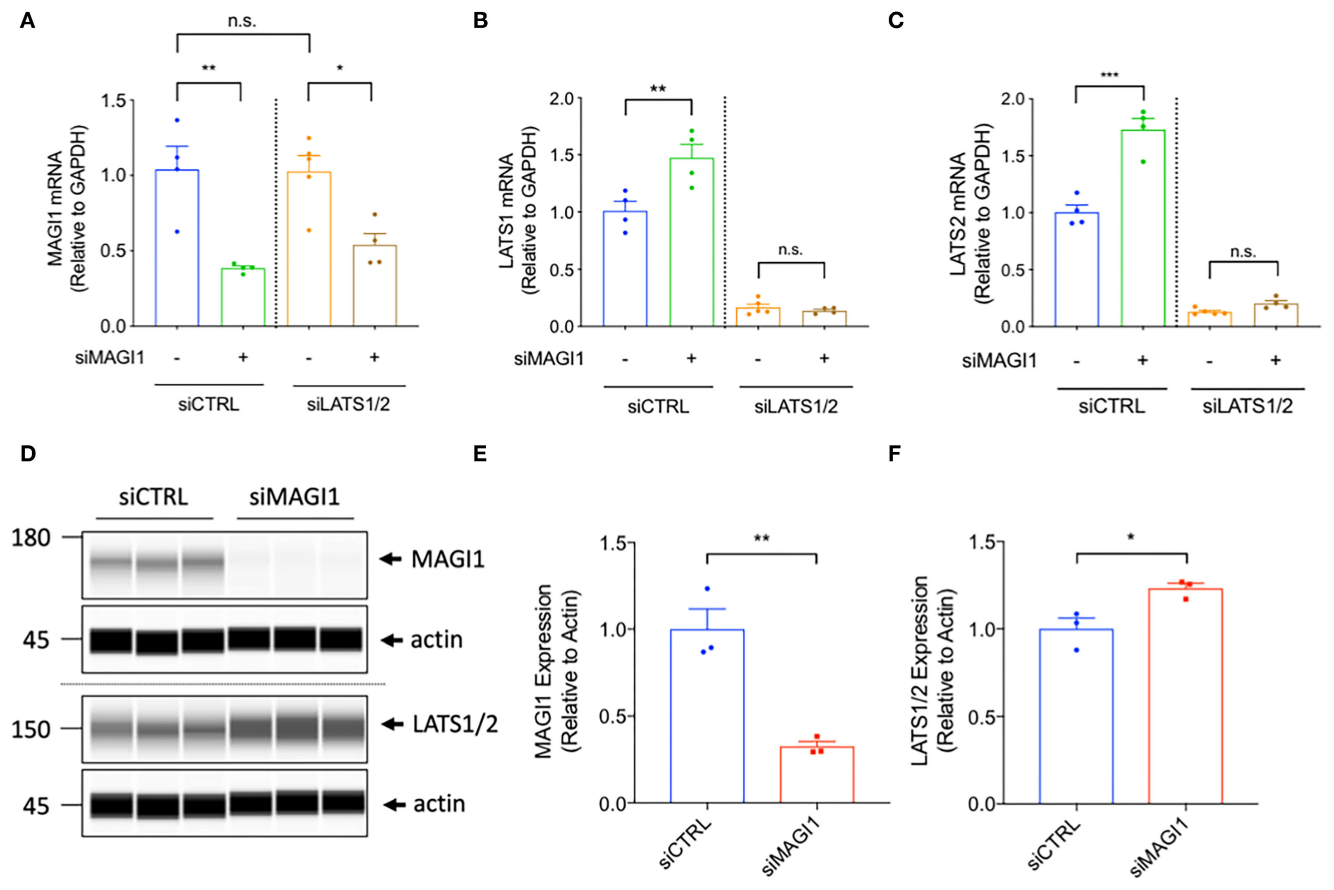
The depletion of MAGI1 increases LATS1/2 expression, but the depletion of LATS1/2 shows no effect on MAGI1 expression. **(A–C)** Levels of MAGI1 and LATS1/2 mRNA expression were quantified in HAECs treated with siMAGI1 with or without siLATS1 and siLATS2, or siCTRL (mean ± SEM, *n* = 4). **(D)** Wes analysis of MAGI1 and LATS1/2 protein expression levels in cell lysates collected from HAECs treated with siMAGI1 (100 nM, 48 h), actin serves as a loading control. **(E)** Quantification of MAGI1 and LATS1/2 expression shown in **(D)** (mean ± SEM, *n* = 3). Statistical differences between two independent groups **(E,F)** were assessed using the Student *t*-test (two-tailed), and one-way analysis of variance followed by Bonferroni *post-hoc* testing for multiple group. ***P < 0.001, **P < 0.01, and *P < 0.05.

The BioID technique and publicly available databases identify MAGI1 as one of the binding partners for LATS (25). This evidence suggests that the MAGI1 WW domains can bind the PPXY motifs on LATS; LATS associates with the adherens junction molecule TRIP6 and colocalizes to cell-cell junctions (18, 39), where LATS can then bind MAGI1 (40). Because MAGI1 can interact with LATS through WW domains and YAP/TAZ can regulate EC barrier function (27), we disrupted the MAGI1-LATS interaction using MAGI1 siRNA to explore the involvement of such interaction in the regulation of EC permeability. Unexpectedly, we found that MAGI1 depletion significantly increases LATS1 and 2 expression both at the mRNA and protein levels (Figure 4). Based on this data, and given the importance of p90RSK in MAGI PTMs, we also examined whether p90RSK affects LATS 1 and 2 expression. By transducing Ad-p90RSK to ECs, we found that p90RSK overexpression does not affect the expression of LATS1 and 2 (Supplementary Figures 1A–C). Furthermore, the overexpression of p90RSK had no effect on the expression and activation of the downstream element YAP (Supplementary Figures 1A,D–F). Since p90RSK activation induces both MAGI1 S741 phosphorylation and K931 deSUMOylation (29), we anticipate that only MAGI1 depletion, but not p90RSK activation or MAGI1 PTMs, regulates LATS1 and 2 expression.

To determine the effect of MAGI1 depletion further downstream of the Hippo pathway, we also detected levels of YAP activation and phosphorylation in MAGI1 depleted ECs. Due to the elevation in LATS1 and 2 expression caused by MAGI1 depletion, we found that YAP phosphorylation (the ratio of p-YAP/total YAP) was accelerated (Supplementary Figures 2A–F). Detection of YAP expression was also inhibited in MAGI1 depleted ECs as a result of this escalation in phosphorylation by LATS1 and 2, further suggesting the decrease of YAP activity (Supplementary Figures 2D–F). To our best knowledge, this is the first report that demonstrates the relationship between decreased MAGI1 expression and increased LATS1 and 2 expression, and subsequent increase of YAP phosphorylation and reduction of YAP expression after the depletion of MAGI1.

### LATS1/2 Promotes EC Monolayer Permeability by Inhibiting YAP

**Figure 5:**
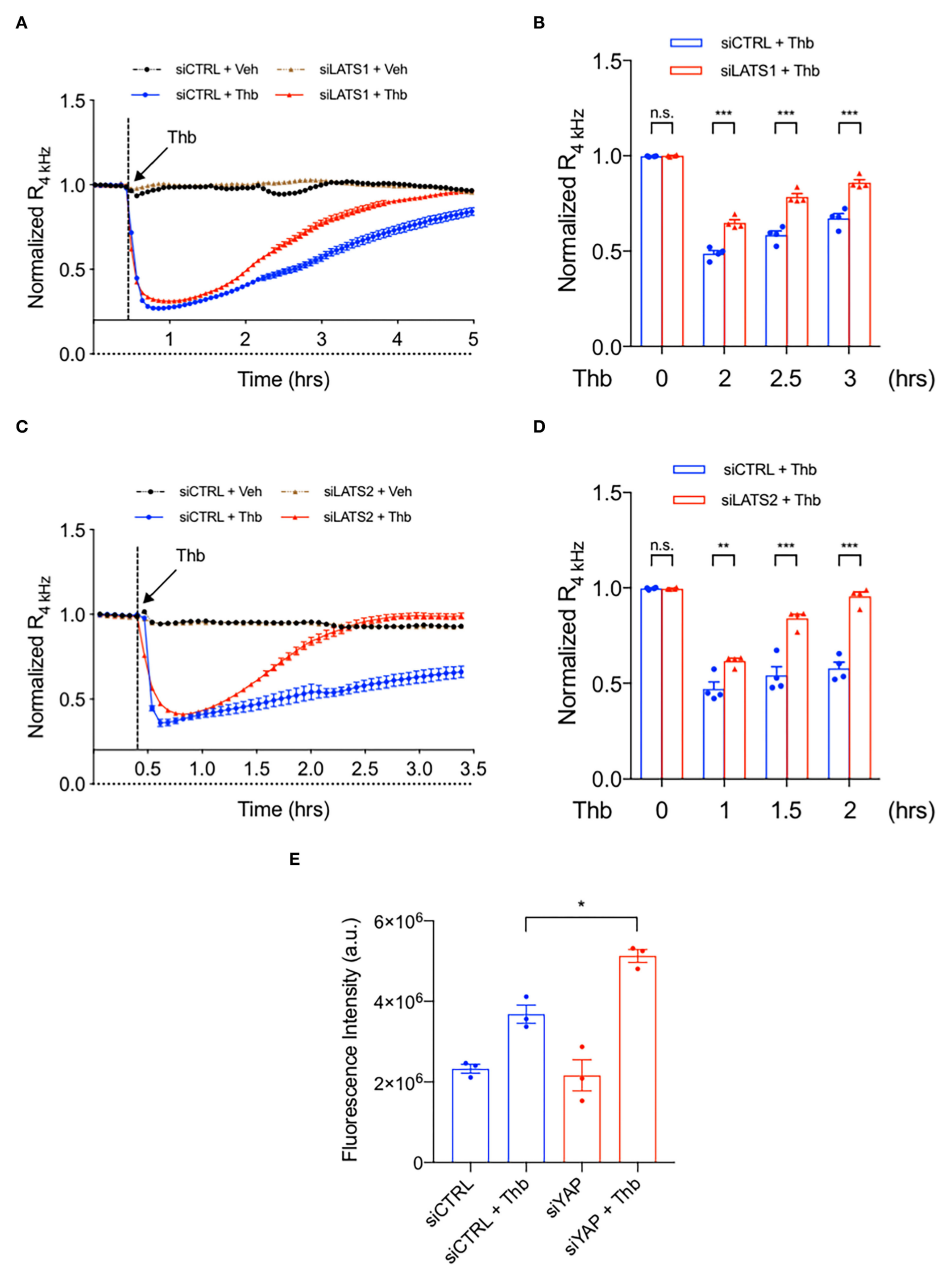
The depletion of LATS1/2 inhibits EC permeability. **(A–D)** Thb (2.5 U/mL)-mediated reduction in TEER values observed in HAECs treated with siCTRL (blue, **A–D**) was inhibited in cells treated with siLATS1 **(A,B)** or siLATS2 **(C,D)**, as assessed by ECIS system and shown as normalized resistance measured approximately every 4 min for indicated times. The dashed line indicates addition of Thb. A reduction in TEER indicates an increase in cell barrier permeability (33) through paracellular mechanisms (34). **(B,D)** Graph demonstrates normalized resistance after Thb treatment at indicated times, relative to basal level (mean ± SEM, *n* = 3–4). Statistical significance was assessed using ANOVA followed by Bonferroni *post-hoc* testing for multiple group comparison. ***P < 0.001 and **P < 0.01. **(E)** Graph demonstrates that Thb (10 U/mL)-induced permeability in HUVECs treated with siCTRL, represented by fluorescence intensity measured in arbitrary units (a.u.), is further increased in HUVECs treated with siYAP (100 nM, 48 h). An increase in fluorescence intensity indicates an increase in cell barrier permeability (35, 36). Statistical significance was assessed using ANOVA followed by Bonferroni *post-hoc* testing for multiple group comparison. *P < 0.05.

**Figure 6:**
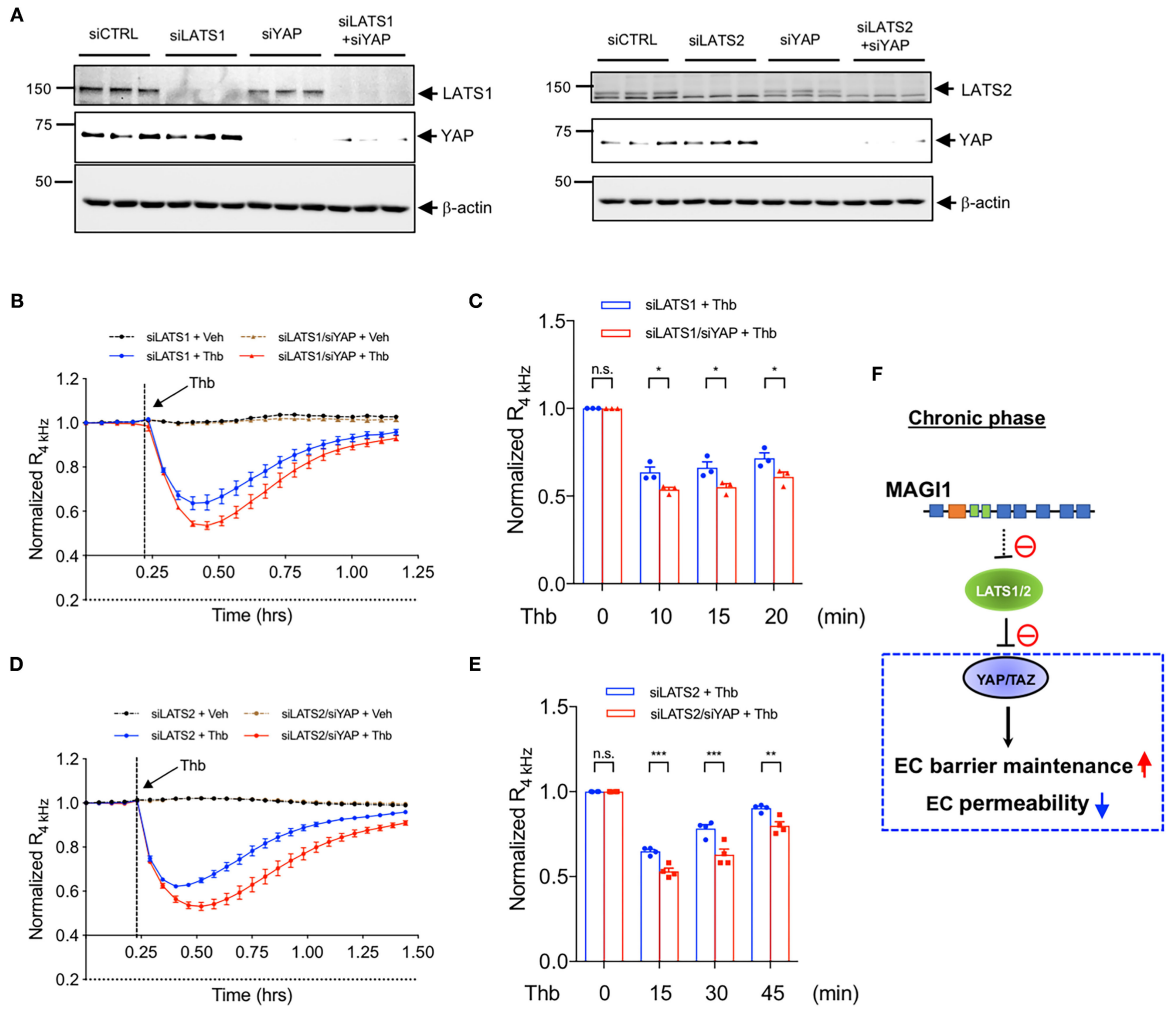
YAP is required in the maintenance of EC barrier function, especially in LATS1/2 depleted condition. **(A)** HAECs were transfected with (Left panel) either siLATS1 or siYAP, or the combination of siLATS1 and siYAP; (Right panel) either siLATS2 or siYAP, or the combination of siLATS2 and siYAP, and IB analyses of LATS1/2 and YAP protein expression levels in resulting cell lysates were performed using anti-LATS1 or 2, anti-YAP, anti-β-actin as indicated **(B–E)**. Thb (2.5 U/mL)-mediated reduction in TEER values observed in cells transfected with siLATS1 (**A** left panel, blue in **B,C**), siLATS2 (**A** right panel, blue in **D,E**) was increased to a greater extent by siYAP transfection, as assessed by the ECIS system and shown as normalized resistance measured approximately every 4 min for indicated times. The dashed line indicates addition of Thb. A reduction in TEER values indicates an increase in cell barrier permeability (33) through paracellular mechanisms (34). **(C,E)** Graph demonstrates normalized resistance after Thb treatment at indicated times, relative to basal level (mean ± SEM, *n* = 3). Statistical significance was assessed using ANOVA followed by Bonferroni *post-hoc* testing for multiple group comparison. ***P < 0.001, **P < 0.01, and *P < 0.05. **(F)** Scheme of MAGI1-mediated Hippo pathway regulation.

To examine if LATS1 and 2 regulate EC permeability, we transfected ECs with either *Lats1* siRNA (siLATS1) or *Lats2* siRNA (siLATS2) and found that the depletion of either LATS1 (Figures 5A,B) or LATS2 (Figures 5C,D) significantly inhibits Thb-induced EC permeability compared to that of the control siRNA (siCont) (Figures 5A–C). Neto et al. have reported the key role of YAP/TAZ in increasing VE-cadherin turnover and junction-associated intermediate lamelipodia, promoting both cell migration, and maintenance of barrier function (27). Lv et al. have also reported that EC permeability is increased in EC specific YAP knock out mice (41). These observations suggest that YAP activation can inhibit EC permeability. To explore this possibility, we depleted YAP using *Yap* siRNA (siYAP) and confirmed that EC permeability is increased in YAP depleted ECs (Figure 5E). Then, we co-transfected siYAP with siLATS1 or siLATS2 to ECs and measured TEER. Note that, a decrease in TEER value indicates an increase in cell barrier permeability (33) through paracellular mechanism (34). The efficacy of these siRNAs was confirmed, as shown in Figure 6A. Compared to cells transfected with siLATS1 (Figures 6B,C; blue line/bars) or siLATS2 (Figures 6D,E; blue line/bars) alone, cells co-transfected with siYAP and siLATS1 or siLATS2 exhibit a greater decrease in TEER (Figures 6B–E; red line/bars), indicating that the reduction of EC permeability mediated by LATS1 and LATS2 is *via* YAP activation, and that YAP is required for the maintenance of EC barrier function (and inhibition of EC permeability) *in vitro*, especially in LATS1/2 depleted condition. We have shown the depletion of MAGI1 upregulated LATS1/2 expression, and inhibited YAP expression (Supplementary Figure 2). Furthermore, we confirmed that siYAP significantly accelerated thrombin-induced EC permeability in Figure 5E. Since we have also shown that the depletion of LATS1/2 inhibited EC permeability via activating YAP (Figure 6), this data suggests that the up-regulation of LATS1/2-mediated inhibition of YAP (Figure 6F) may contribute to the part of the depletion of MAGI1-mediated acceleration of EC permeability, although further experimentation is necessary to be certain of this relationship.

### p90RSK Specific Inhibitor, FMK-MEA, Reduces Tumor Vessel Hyper-Permeability Without Altering Tumor Vessel Morphology

**Figure 7:**
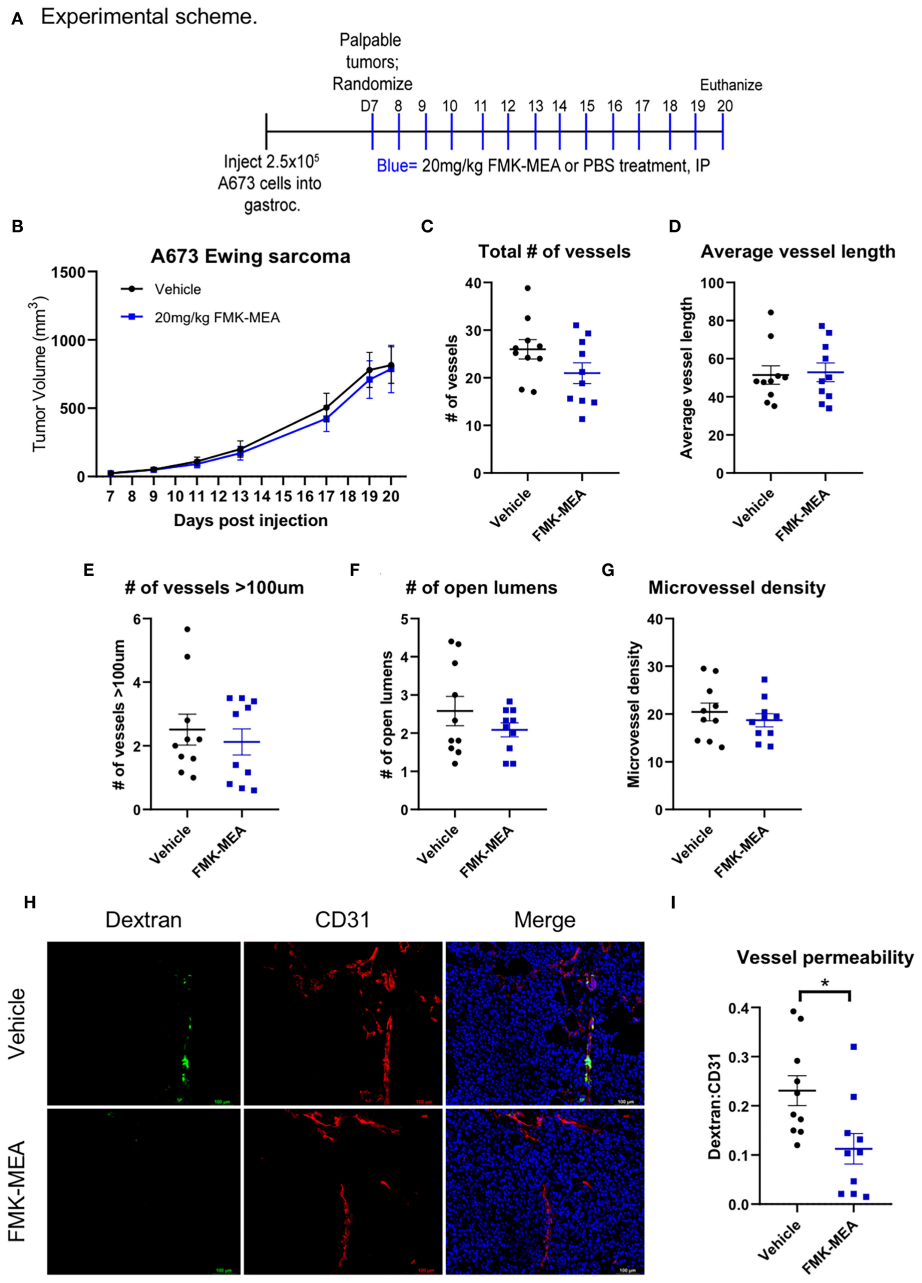
Pharmacological inhibition of p90RSK reduces tumor vessel permeability without altering vascular structure or tumor growth. **(A)** Experimental scheme of mouse experiment. **(B)** A673 Ewing sarcoma orthotopic tumor volumes were plotted over time (means ± SEM, *n* = 10 mice/treatment). **(C–F)** Tumor vasculature structural analysis was performed by quantifying number of vessels, average vessel length, number of vessels >100 um, number of open lumens, and microvessel density in 5 random fields/tumor (means ± SEM, *n* = 10/group). Statistical significance was assessed using student's *t*-test. **(G)** Representative images of FITC-dextran (green), CD31 (red), and DAPI (blue) immunofluorescence. **(H,I)** Tumor vessel permeability was compared between treatment groups using dextran:CD31 quantification (means ± SEM, *n* = 10/group). Statistical significance was assessed using student's *t*-test. *P < 0.05, **P < 0.01, ***P < 0.001.

Overexpression of p90RSK increases EC permeability *in vitro*, while both p90RSK specific inhibitor FMK-MEA and the dominant negative form of p90RSK decreases it (Figures 1A–F). Thus, we investigated the effect of p90RSK activation on EC permeability *in vivo* using FMK-MEA. In this study, we aimed to determine the effect of FMK-MEA on p90RSK-mediated EC leakiness, independent of its effect on tumor growth. Therefore, we chose a relatively low dose of FMK-MEA 20 mg/kg/day (42), and treated A673 Ewing sarcoma tumor-bearing mice with FMK-MEA as shown in Figure 7A. To evaluate the effect of FMK-MEA on the structure of the tumor vasculature, we stained frozen tissue sections obtained from the A673 tumor bearing mice with anti-CD31 antibody (Figures 7C–I). We noted no significant difference in tumor size (Figure 7B), the number of total vessels (Figure 7C), vessel length (Figure 7D), the number of vessels with >100 μm in diameter (Figure 7E), the number of open lumens (Figure 7F), or microvessel densities (Figure 7G) between tumors from control or FMK-MEA treated mice. Strikingly, although there were no significant changes in the morphology of the tumor vasculature between the two groups, there was a significantly lower proportion of hyper-permeable tumor vessels in the FMK-MEA treated group, assessed by high molecular weight dextran leakage (Figures 7H,I).

**Figure 8:**
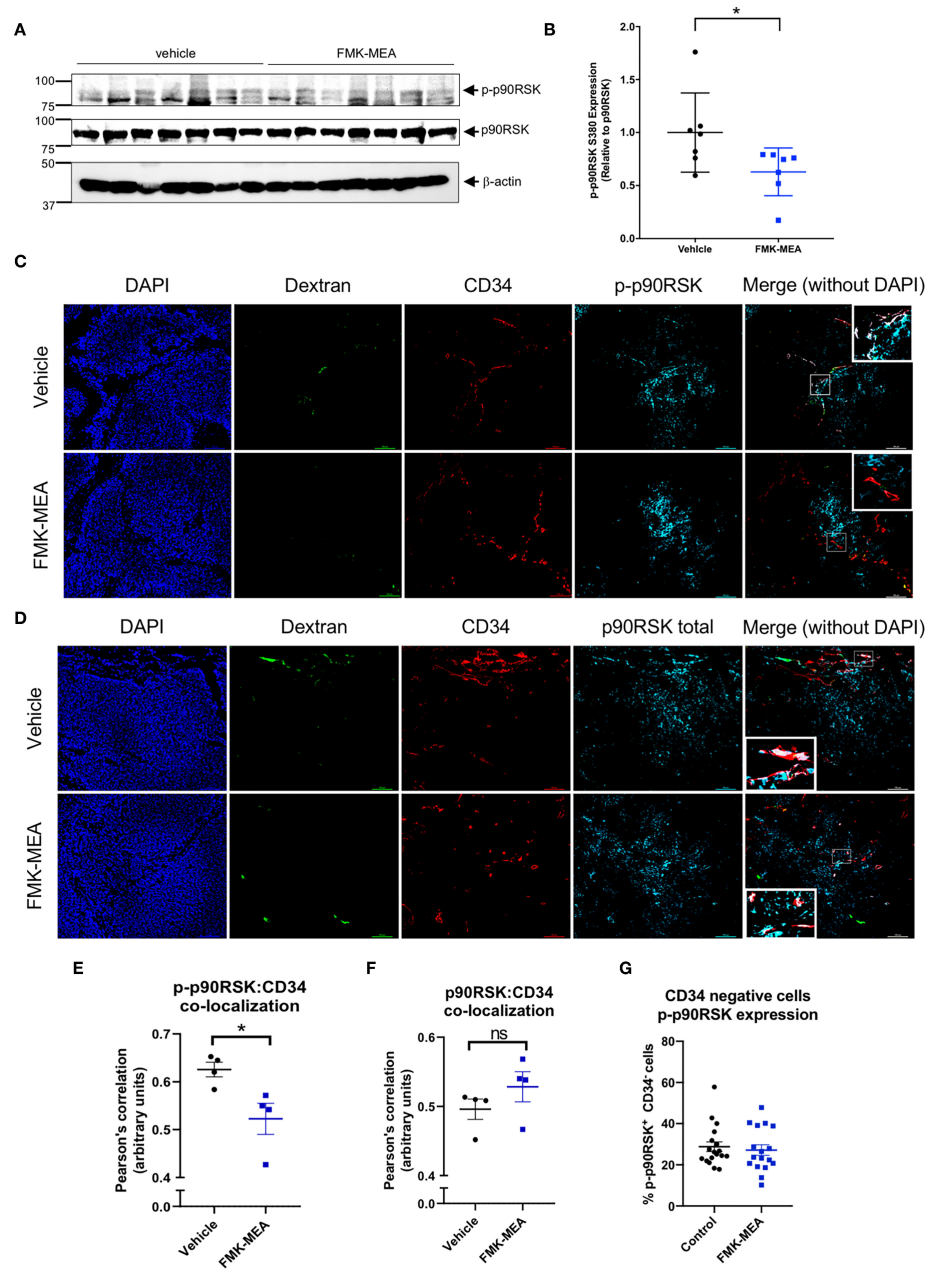
FMK-MEA reduces p90RSK activation in CD34^+^ but not CD34^−^ cells in A673 Ewing sarcoma orthotopic tumor. **(A)** After 20 days of injection of A673 Ewing sarcoma cells as described in Figure 7A, tumors were isolated and IB analysis of p90RSK phosphorylation, p90RSK protein expression level in resulted cell lysates was performed using anti-p-p90RSK, p90RSK, and b-actin antibodies as indicated (left). **(B)** Graph shows densitometric quantification of phosphorylated p90RSK, which was normalized by total p90RSK protein levels. Data represent mean ± S.D., *n* = 7, *p < 0.05, **(C,D)** Representative images of FITC-dextran (green), CD34 (red), p-p90RSK **(C)**, or p90RSK **(D)** (blue) in A673 Ewing sarcoma tumor samples. **(E,F)** Co-localization between CD34 and p-p90RSK **(E)** or p90RSK **(F)** staining was quantified in 5 random fields/tumor using Pearson's correlation coefficient. Data represent mean ± S.D., *n* = 4, *p < 0.05, **(G)** Around 100–200 cells of CD34 negative but p-p90RSK positive or negative cells were counted from 5 random fields/tumor, and ratios per all counted cells by CD34 negative were calculated. Data represent mean ± S.D. N.S. = not significant. All the statistical analyses were performed by unpaired 2-tailed *t*-test.

When p90RSK phosphorylation was evaluated by western blot on whole tumor lysates, we found that FMK-MEA marginally inhibits p90RSK phosphorylation relative to control (Figures 8A,B). Based on antibody specifications we stained formalin-fixed, paraffin embedded tissue sections for CD34 (Figures 8C–F). CD34 is often used as a marker for tumor vasculature in cancer studies (43, 44), and CD34^+^ cells have been used to characterize vascular patterns within tumor tissues (45, 46). We found FMK-MEA significantly inhibits p90RSK phosphorylation in tumor vasculature, as shown by the increased Pearson's correlation between CD34^+^ cells and phosphorylated-p90RSK^+^ cells (Figures 8C,E). The level of total p90RSK expression was unchanged by FMK-MEA treatment (Figures 8D,F). Of note, the Pearson's correlation between CD34^−^ (i.e., non-endothelial) cells and phosphorylated-p90RSK^+^ cells is not different between the two groups (Figures 8C,G). These data suggest that FMK-MEA at the dose of 20 mg/kg/day is unable to inhibit the activation of p90RSK in tumor cells (Figures 8C,G) but is able to prevent the activation of p90RSK in the tumor vessels, and that p90RSK activation is crucial in the regulation of tumor vasculature hyper-permeability.

## Discussion

Reversible cell contraction and inter-endothelial gap formation induced by certain inflammatory mediators are accompanied by increased endothelial barrier permeability (47). In our previous study, we reported the role of p90RSK-mediated MAGI1 S741 phosphorylation and K931 de-SUMOylation in Thb-induced inflammatory responses (38). In the current study, first we found that MAGI1 S741 phosphorylation and K931 de-SUMOylation, both mediated by p90RSK activation, play a major role in disrupting endothelial barrier function. Next we revealed the importance of p90RSK activation in tumor vessel hyper-permeability by demonstrating that treatment of tumor bearing mice with the p90RSK specific inhibitor, FMK-MEA, significantly inhibits tumor vessel permeability without affecting tumor vessel morphology. We also found that MAGI1 inhibits LATS1/2 expression and maintains EC barrier function by regulating the Hippo pathway. This data provides insights into the role of MAGI1 in the regulation of EC permeability, not only by PTMs, but also by inhibiting the Hippo pathway. Taken together, this data suggests the crucial role of the p90RSK-MAGI1 pathway in promoting both EC activation and permeability in concert via PTMs and transcriptional regulation of the Hippo pathway.

EC permeability is controlled by both tight and adherens junctions, which seal the vessel's inner surface between opposing ECs (48). MAGI1 localizes to both tight and adherens junctions (49). We have reported that MAGI1 K931 de-SUMOylation following Thb stimulation of ECs is crucial for nuclear translocation of MAGI1 (38). Consistent with this result is that the MAGI1 K931R mutant, which cannot be SUMOylated, dissociates endogenous MAGI1 from tight and adherens junctions to translocate to the nucleus and promotes EC permeability. MAGI1 K931 de-SUMOylation is dependent on SENP2 T368 phosphorylation and subsequent SENP2 nuclear export. Therefore, our current data suggests that the translocation of SENP2 between the cytosol and the nucleus can regulate the nuclear and cytosolic/membrane events in concert, and dynamically regulate both EC permeability and activation. This would be critical because the coordination of adhesion molecule expression with EC barrier opening is necessary for leukocyte and monocyte infiltration into damaged vessels. The current study indicates the role of the p90RSK-MAGI1 module in this process.

Evidence suggests a role for the Hippo pathway in regulating adherens junction function. Dutta et al. showed that TRIP6 associates with LATS1 and 2 and inhibits LATS1 and 2 activation (40). Chastre et al. reported that MAGI1 directly interacts with TRIP6 (18). These observations suggest that MAGI1 can interact with LATS1 and 2, and that the MAGI1-LATS1 and 2 interaction may play a role in EC barrier function. In this study, we found that the depletion of MAGI1 increases LATS1 and 2 expression at both mRNA and protein levels. Neto et al., have reported a key role of YAP/TAZ in increasing VE-cadherin turnover and junction-associated intermediate lamelipodia, promoting both cell migration and maintenance of barrier function (27). As shown in Figure 6, reduction of EC permeability induced by depletion of either LATS1 or LATS2 was reversed in cells co-transfected with YAP siRNA, suggesting that the reduction of EC permeability induced by LATS1 and LATS2 is due to YAP activation. Consistent with this observation, a reduction in BMP signaling induced by YAP/TAZ activation has been suggested to mediate EC barrier function maintenance (27).

The transcription of the LATS2 gene can be directly regulated by transcription factors p53 and FOXP3 (50–55). Interestingly, YAP/TAZ and their canonical transcriptional factor TEAD can directly increase LATS2 transcription and form a negative feedback loop (53–55), shutting down the duration of YAP activity and attenuating for the excessive YAP activation and the consequent oncogenic effects. The transcription of the LATS1 gene can be positively regulated by transcriptional factor CUX1, which accelerates S-phase and tumorigenesis (56). In several cancer types, the increase of LATS1 and 2 has been reported, and the expression levels are correlated with invasive and migratory capacities (57–59). These observations suggest that LATS1 and 2 not only play a role as tumor suppressors but also can also promote tumorigenesis, especially when LATS expression is continuously high. Although LATS1 and 2 and YAP/TAZ signaling has been implicated in tumorigenesis, these findings are largely based on experimental models which modulate LATS1 and 2 and/or YAP/TAZ signaling within the tumor cell, not within the endothelium. The role of these proteins within tumor endothelium is not well-understood in the context of tumorigenesis. Our data suggests that MAGI1 regulates LATS1 and 2 expression transcriptionally, and that MAGI-mediated reduction of LATS1 and 2 expression leads to increased YAP activation, which is critically involved in the maintenance of EC barrier function (Figure 6F). Note that we cannot exclude the possibility that MAGI1 can post-translationally regulate LATS1 and 2 expression. The increase of LATS1 mRNA induced by the combination of HGF and VEGF stimulation in ECs has been suggested but the regulatory mechanism of LATS1 and 2 mRNA expression remains unclear (60). As such, additional investigation is warranted to examine how MAGI1 can inhibit LATS1 and 2 expression.

Neto et al. have suggested that the activation of YAP inhibited EC permeability by suppressing the expression of Notch and BMP target genes transcriptionally (chronic phase, Supplementary Figure 4) (27). This suggests that Thb-induced transcriptional YAP activation plays a minimum contribution to the Thb-induced EC permeability during the acute phase within 30 min after Thb stimulation, before expression of Notch and BMP genes is increased. Together with our data, this is consistent with a model in which MAGI1-LATS1/2-YAP signaling plays a role during the chronic phase only, which is regulated by the protein expression level, and not by phosphorylation or SUMOylation level.

Previously, we found that MAGI1 S741 phosphorylation increases Rap1 activation, which localizes in the cytosol and upregulates NF-kB activation and adhesion molecules expression in ECs (38). However, the role of MAGI1 S741 phosphorylation in nuclear translocation remains unclear, and p90RSK regulates both MAGI1 phosphorylation and de-SUMOylation independently (38). Interestingly, we found that ECs expressing the MAGI1 S741 phosphorylation resistant mutant inhibited vascular permeability. Since Rap1 activation is known to decrease endothelial permeability, the increased endothelial permeability by MAGI1 S741 phosphorylation cannot be explained by Rap1 activation (61). It is possible that MAGI1 S741 phosphorylation can change the interaction of other junctional proteins such as JAM4 and increase endothelial permeability. Thus, further investigation will be necessary.

Finally, we demonstrated that p90RSK signaling is a mediator of tumor vascular hyper-permeability, which has important implications for the efficacy of several standard of care cancer treatments. Thus, the signaling mechanisms that we have identified in this work should be evaluated in future work to determine whether modulation of p90RSK-MAGI1 and MAGI1 mediated signaling has therapeutic potential in cancer models.

The purpose of our TEER studies was to show that ECs in which certain signaling proteins were depleted respond differently to thrombin (Tb). Although we standardized the cell culture condition (such as cell plating condition and pre-culture period), we observed considerable variation among the baseline TEER values. This is likely to be due to different tightness of monolayers, which cannot be detected by morphology but can be detected by TEER measurements. However, when we compared the baseline TEER values of monolayers of ECs treated with and without various adenovirus vectors, there was no statistically significant difference between Ad-MAGI1-WT and other MAGI1 mutants (Supplementary Figure 3), suggesting that MAGI1 mutations do not affect the baseline TEER. When monolayers of cells were treated with Thb, the direction (increase or decrease) and extent of TEER changes were consistent within cells with the same adenoviral MAGI1 (for example, cells with Ad-WT-MAGI1 or with Ad-MAGI1-KR). However, if we statistically compare the end-point TEER values with the baseline values, we often did not see difference. This is due to large variations in both the baseline and the end-point values.

Of note, the basal difference of EC permeability without Thb stimulation reflects not only tight and adherens junction organization and function, but also many other cellular events including proliferation, migration, and apoptosis (chronic phase, Supplementary Figure 4), which are very different from the molecular mechanisms involved in Thb-induced EC permeability (acute phase, Supplementary Figure 4) (62–64). Therefore, we may detect some different response of EC permeability at the basal level (chronic phase) compared to the response after Thb stimulation (acute phase, Supplementary Figure 4). For example, we found a small increase of EC permeability by Ad-DN-p90RSK transduction at basal level (*p* = 0.048) (Supplementary Figure 3B). Since inhibition of p90RSK can inhibit EC proliferation, this may lead to the small increase of EC permeability induced by DN-p90RSK. Also we found the small increase of EC permeability after siLATS2 transfection (*p* = 0.03) (Supplementary Figure 3D). The depletion of LATS2 can increase YAP activation. YAP activation can increase EC inflammation, which may increase EC permeability. Therefore, this chronic phase mechanism can be very different from acute phase of Thb-induced EC permeability. Since the difference of these EC permeability difference is very small at chronic phase, to determine the molecular mechanism for this small EC permeability difference at basal level would be difficult.

ECIS has only 16 chambers, and we need two conditions of vehicle and Thb stimulation, so we only have eight chamber for each group. Therefore, we cannot compare 3–4 conditions (siCont, siYAP, siLATS1, siLATS1/siYAP) by triplicates in one experiment. Therefore, we used siLATS1 or siLAT2 as an internal control between Figures 5, 6. Our data of siYAP is consistent with the previous reports, and the down-regulation of LATS1/2 can increase YAP transcriptional activation is well-established. Therefore, it is fair to conclude that the siLATS1/2 inhibits Thb-induced EC permeability (Figure 5) is, at least partially, due to YAP activation (Figure 6).

## Materials and Methods

### Animal Study

Animal experiment was approved by MD Anderson's Institutional Animal Care and Use Committee and adhered to National Institutes of Health standards. Six weeks-old male nude mice were obtained from the Experimental Radiation Oncology Breeding Core at MD Anderson. A673 Ewing sarcoma tumor cells (2.5 × 10^5^) in 100 μL of phosphate-buffered saline (PBS) were injected intramuscularly into the gastrocnemius. When tumors were ~35–50 mm^3^ (9 days after injection), the mice were divided into the following two cohorts of 10 mice each with equal average tumor sizes: PBS (Vehicle) or 20 mg/kg FMK-MEA administered intraperitoneal once per day for 12 consecutive days. All mice were euthanized 20 days following tumor cell inoculation. All mice received an intravenous injection of 1 mg of high molecular weight (2 × 10^6^ kDa) dextran-fluorescein isothiocyanate (FITC; Sigma-Aldrich) into the lateral tail vein 5 min prior to euthanasia. Tumors were harvested and fixed in formalin, frozen in optimal cutting temperature (OCT) compound, or snap frozen for protein analysis.

### Immunofluorescent Staining of Frozen Tissue Sections

Frozen tumor sections were fixed in ice-cold 4% paraformaldehyde (PFA) for 10 min. Slides were washed with PBS and then incubated with 4% fish gel solution for 1 h at room temperature. Primary antibody (rat anti-mouse CD31, BD Bioscience 553370, 1:50 dilution) was then placed on slides and stored at 4°C overnight. Slides were then washed with PBS and incubated with secondary antibody (goat anti-rat Texas Red, 1;1000 dilution, ThermoFisher T6392) for 1 h at room temperature. Nuclei were stained with Fluoro-Gel II with DAPI (Electron Microscopy Sciences, Cat # 17985). Images were captured with a Nikon Eclipse Ti de-convolution inverted fluorescent microscope and analyzed using NIS-Elements Imaging Software.

### Evaluation of the Structure and Leakiness of Tumor Vasculature

The structure of tumor vasculature was evaluated by measuring the areas of CD31-positive structures (microvessel density) and counting the numbers of visible lumens, total vessels, and vessels with >100 μm in diameter. The number of lumens or vessels was counted in five random 20× magnification photographs per each slide and was averaged to obtain an average value for each tumor (Figures 7C–G). For dextran analysis, the number of dextran-positive vessels was normalized to the number of CD31-positive vessels (Figure 7I) in five random 20× magnification photographs per each slide and was averaged to obtain an average value for each tumor. Photographs were taken and analyzed in a blinded fashion without knowledge of the treatment group.

### Immunofluorescent Staining of Paraffin-Embedded Tissue Sections

Paraffin-embedded slides were deparaffinized using xylene and ethanols. Antigen retrieval was performed using sodium citrate buffer, pH 6.0 containing 0.05% Tween-20. Blocking solution containing 10% goat serum diluted in Dako antibody diluent (Dako, Code S0809) were placed on slides for 1 h at room temperature. Primary antibody including phospho-p90RSK (Cell Signaling, 9341S), or CD34 (Abcam, ab8158) were used at 1:100 dilution and total p90RSK1 (Abclonal, A157118) at 1:50 dilution. 1:1000 goat anti-rabbit Alexa Fluor 647 (ThermoFisher, A27040) and 1:1000 goat anti-rat Texas Red (ThermoFisher, T6392) were used as secondary antibodies.

Images were captured with a Nikon Eclipse Ti de-convolution inverted fluorescent microscope.

**Co-localization of fluorescence** was quantified using Pearson's correlation coefficient in five random 20× magnification photographs per each slide and was averaged to obtain an average value for each tumor (Figures 8E,F).

### Quantification of p90RSK Phosphorylation in Tumor Cells

Five random 20x magnification pictures were obtained from four tumor samples collected from mice treated with vehicle and four tumor samples collected from mice treated with FMK-MEA. Four random fields (100 × 100 um) that contain tumor cells was selected as region of interest (ROI) in each picture. Cells that are p-p90RSK positive and CD34 negative (non-tumor vasculature cells with p90RSK activation) and total CD34 negative cells (total non-tumor vasculature cells) were counted. Then, the number of non-tumor vasculature cells with p90RSK activation was divided by the number of total non-tumor vasculature cells to obtain the percentage of non-tumor vasculature cells with p90RSK activation per 100 × 100 um ROI. This percentage is reported in Figure 8G.

### Antibodies, Materials, and Reagents

A rabbit antibody against phosphorylated MAGI1 S741 was produced by Pierce Biotechnology Inc. (Rockford, IL, USA) using a peptide corresponding to amino acids 735−748 of the human MAGI1 sequence (Ac-PLERKDS^*^QNSSQH-C). This peptide was synthesized and was immunized to rabbits at 1:1 ratio of saline and adjuvant (maximum 1 mL was used). The initial inoculation was done via subcutaneous routes, and following injections were spread out into a minimum of four different sites to avoid any lesion formation. After the initial inoculation, the rabbits were checked for any adverse reactions including lesion formation, loss of appetite, and non-responsiveness. Once the rabbits have passed the initial evaluation, the remaining immunizations were done at week 2, 4, 6, 12, 18, 21, and 28 after the initial inoculation. At each repeated immunization, the serum collection was performed and tested by Elisa. At the end of immunization process, the rabbits were terminated and the obtained sera were affinity purified. All other antibodies used in this work were commercially obtained, and were listed in the Table 1. Human alpha-thrombin (Thb) was purchased from Haematologic Technologies (cat. no. HCT-0020; Essex Junction, VT, USA). Small interfering RNA (siRNA) targeting human MAGI1 corresponding to nucleotides 843–857 of the coding sequence 5′-GGACCCUUCUCAGAAGUUCCCUCAA (13) was purchased from Thermo Fisher Scientific (Waltham, MA, USA) and Sigma (St. Louis, MO, USA). siRNA targeting human LATS1 (#L-004632-00-0005; (1) GGUGAAGUCUGUCUAGCAA; (2) UAGCAUGGAUUUCAGUAAU; (3) GGUAGUUCGUCUAUAUUAU; (4) GAAUGGUACUGGACAAACU) and LATS2 (L-003865-00-0005; (1) GCACGCAUUUUACGAAUUC; (2) ACACUCACCUCGCCCAAUA; (3) AAUCAGAUAUUCCUUGUUG; (4) GAAGUGAACCGGCAAAUGC) were purchased from GE Healthcare Dharmacon. Non-specific siRNA negative control was purchased from Invitrogen (AM4636).

**Table 1:** The information of antibodies used in this work.

**Antibodies**	**Companies**	**Catalog number**
p90RSK	Novus Biologicals	MAB2056
p-p90RSK	Cell Signaling	9341S
MAGI1	Santa Cruz Biotechnology	sc-100326
Tubulin	Sigma-Aldrich	T9026-.2ML
LATS1/2	MyBioSource	MBS8241758
LATS1	Bethyl	A300-478A-M
LATS2	Abcam	Ab110780
YAP	Cell Signaling	4912S
p-YAP S127	Cell Signaling	4911S
Mouse anti β-actin	Novus Biologicals	NBP1-47423
Rabbit anti β-actin	Novus Biologicals	NB600-532

### Plasmids and Adenoviruses

EGFP-MAGI1 construct (The GenBank gene accession number: KY651081) was a kind gift from Dr. Mochizuki (Department of Cell Biology, National Cardiovascular Center Research Institute, Osaka, Japan) (13). pCMV-FLAG-tagged-MAGI1-WT was generated by sub-cloning MAGI1 from EGFP-MAGI1 into the pCMV-Tag2B vector (Agilent Technologies, Santa Clara, CA, USA) at sites recognized by restriction enzymes *Eco*RI and *Hin*dIII. pCMV-FLAG-tagged-MAGI1-S741A mutant was generated by site-directed mutated pCMV- FLAG-tagged-MAGI1-WT using a QuikChange site-directed mutagenesis kit (Agilent Technologies) according to the manufacturer's instructions. FLAG-tagged adenoviral vectors containing MAGI1-WT and -S741A mutant were generated by cloning each corresponding insert from pCMV- FLAG-tagged-MAGI1-WT and -S741A into the pENTR1A vector (Life Technologies) at sites recognized by the restriction enzymes *Kpn*I and *Not*I, and then a recombinase reaction was performed to get a pDEST-based vector following manufacture's instruction (#K4930-00, ViraPower Adenoviral Expression System, Promega). Where indicated, an adenovirus containing β-galactosidase (Ad-LacZ) was used as a control (42).

### Cells

Human umbilical vein endothelial cells (HUVECs) were obtained from collagenase-digested umbilical cord veins (65) and collected in M200 medium supplemented with LSGS (Cascade Biologics, Inc., Portland, OR) and 2% FBS (Atlanta Biologicals, Inc., Lawrenceville, GA). Human aortic endothelial cells (HAECs) were a kind gift from Dr. Lusis (UCLA, David Geffen School of Medicine). HUVECs and HAECs were cultured in Petri dishes or flasks coated with 0.2% gelatin type A (cat. no. 901771; MP Biomedicals, Santa Ana, CA, USA), in Endothelial Cell Medium (ECM, Cat.no. 1001, ScienCell, Carlsbard, CA. USA) containing 465 mL of basal medium, 25 mL of fetal bovine serum (FBS, Cat. no. 0025, ScienCell, Carlsbard, CA, USA), 5 mL of Endothelial Cell Growth Supplement (ECGS, Cat. no. 1052, ScienCell, Carlsbard, CA, USA) and 5 mL of penicillin/streptomycin solution (P/S, Cat. no. 0503, ScienCell, Carlsbard, CA, USA). Only HAECs with <15 passages were used in this study.

### SDS/PAGE and Immuno-Blotting (IB)

At the end of experiments, ECs were washed three times in ice-cold PBS and lysed by adding a sufficient volume of 1X cell lysis buffer (cat. no. 9803S; Cell Signaling Technology, Danvers, MA, USA) or modified RIPA buffer (50 mM Tris-HCl, pH 7.4, 150 mM NaCl, 1 mM ethylenediaminetetraacetic acid, 1% Nonidet P-40, 0.1% sodium dodecyl sulfate (SDS), 0.25% sodium deoxycholate) supplemented with a mammalian protease inhibitor cocktail (cat. no. p8340; Sigma, St. Louis, MO, USA), 1 mM phenylmethylsulfonyl fluoride (cat. no. 36978; Thermo Fisher Scientific, Waltham, MA, USA), and 20 mM N-ethylmaleimide (cat. no. E3876; Sigma, St. Louis, MO, USA). The resulted cell lysates were centrifuged at 15,000 rpm for 15 min, and supernatants were collected. Protein concentrations were determined using a standard BCA protein assay. For IB, we loaded equal protein amounts from control and treated samples in each well of SDS-polyacrylamide gel, and proteins were resolved using SDS-polyacrylamide gel electrophoresis and electro-transferred onto Immobilon polyvinylidene fluoride transfer membranes (cat. no. IPVH00010; EMD Millipore, Darmstadt, Germany). The membranes were then immunoblotted with an antibody against each indicated protein. We incubated with the primary antibodies at 1:1000 and at 1:5000 dilutions for goat anti-mouse or anti-rabbit secondary antibodies conjugated with HRP. Resulted membranes were visualized using an enhanced chemiluminescence detection reagent (cat. no. 170-5060; Bio-Rad, Hercules, CA, USA) following the manufacturer's instructions.

### Automated Capillary Electrophoresis Western Analysis (WES)

Whole cell lysates were collected in modified RIPA buffer as described in the IP and IB section. A total of 5 μL of 0.4-1 mg/mL protein was loaded into plates and capillary electrophoresis western analysis was carried out following the manufacturer's instructions (Protein simple WES, part no. 004-600, ProteinSimple, San Jose, CA) using the 12–230 kDa Separation Module (part no. SM-W003, ProteinSimple, San Jose, CA) and either Rabbit (part no. DM-001, ProteinSimple, San Jose, CA) or Mouse (part no. DM-002, ProteinSimple, San Jose, CA) Detection Modules. Briefly, whole cell lysates were mixed with 5X fluorescent master mix containing 200 mM DTT followed by heating at 95°C for 5 min. Cell lysates, blocking buffer (antibody diluent), primary antibodies (in antibody diluent), HRP-conjugated secondary antibodies, and luminol-peroxide were then dispensed onto the separation plate. Antibodies against β-actin served as loading controls and were multiplexed with the primary antibodies for all samples. Capillary electrophoresis was performed using the instrument default settings: separation time 25 min, separation voltage 375 V, blocking 5 min, primary and secondary antibodies 30 min. Finally, automatically detected standards and peaks were manually inspected, and the data were analyzed with the inbuilt Compass software (ProteinSimple) (66).

### Assessment of Barrier Function by Trans-Endothelial Electrical Resistance (TEER) Measurements and Trans-Endothelial Permeability Assay

TEER values of EC monolayers grown on the surface of small and planar gold-film-electrodes plated on the bottom of 8W10E+ array chambers treated with Thb was measured at low frequency (4,000 Hz), at which the membrane impedance increases, and more current find an easier route going under and between the cells. The measurement was performed in real-time by electric cell-substrate impedance sensing (ECIS) based system. Briefly, the 8W10E+ array chambers were treated with 10 mM L-Cysteine solution (room temperature, 15 min) followed by washing twice with ultra-pure water. The treated chambers were then coated with 0.2% gelatin type A. ECs were seeded into the chambers and grown in complete ECM overnight to produce a confluent monolayer. Next day, baseline resistance measurements were taken. Upon stabilization, Thb was added, and change in TEER values were recorded by an ECIS-Zθ instrument (Applied BioPhysics Inc., Troy, NY, USA) connected with a Dell personal computer equipped with ECIS software (Applied Biophysics). Figures illustrate normalized TEER values (where the value of 1.0 represents the basal TEER measurement immediately before adding Thb). A reduction in TEER values indicates an increase in cell barrier permeability (33) through paracellular mechanisms (34). By comparison, the response of LATS and MAGI1 depletion to Thb (Figures 5, 2E) are on a different time scale than that of MAGI1 overexpression (Figure 2B), which may be due to transfection methods: we induced the depletion of LATS or MAGI1 (Figures 1A, 2E, 5, 6) by transfecting LATS siRNA or MAGI1 siRNA using the Plus/Lipofectamine 2000 mixture; we transiently overexpressed MAGI1by transducing ECs with adenovirus expressing MAGI1 (Figures 1B, 2B, 3). The use of the transfection mixture composed of Plus and Lipofectamine 2000 reagent may cause some EC damage, which slows down the response to Thb. Therefore, we cannot compare the different kinetics of responses between siRNA transfection and adenovirus transduction.

As indicated in the figure legends, in some cases, following appropriate treatments, ECs were seeded onto ThinCerts™ 0.4 μm translucent (12-well; Greiner, UK) and grown to confluence. When needed, ECs were pre-treated with p90RSK specific inhibitor FMK-MEA prior to stimulation with Thb. ECs were then washed in DPBS at room temperature and 1 mg/ml FITC-dextran 4 kDa (Sigma, Poole, UK) diluted in phenol red free EBM supplemented with 1% (v/v) FCS was added and incubated for 25 min at room temperature. The fluorescence level in the flow through was measured on a plate reader (Ex 490 nm and Em 525 nm). An increase in fluorescence intensity measured in arbitrary units (a.u.) indicates an increase in cell barrier permeability (35, 36).

## siRNA and Adenovirus Treatment

For LATS siRNA treatment, cells were incubated with the mixture of LATS1 siRNA (siLATS1: sense 5′-cggcaagauagcauggauuucagua 3′ and anti-sense: 5′-uacugaaauccaugcuaucuugccg 3′, final concentration 100 nM) and LATS2 siRNA (siLATS2: sense 5′-cuuuaaccuguuguaauuagacucu, and anti-sense 5′-agagucuaauuacaacagguuaaag, final concentration 100 nM) in a mixture of Plus and Lipofectamine 2000 transfection reagent at the ratio of Plus: Lipofectamine 2000 = (1:1.5). After transfection, cells were allowed to recover in the complete medium for 48 h (42). After transfection, cells were allowed to recover in the complete medium for 24–48 h. For adenoviral transduction, we used 20 multiplicities of infection (MOI).

### Statistics

Differences between two independent groups were determined using the student's *t*-test (two-tailed). Differences between multiple groups were determined using one-way analysis of variance (ANOVA) followed by Bonferroni *post-hoc* testing for multiple group comparison by GraphPad Prism (GraphPad Software, San Diego, CA, USA). *P* < 0.05 were considered statistically significant and are indicated by one asterisk in the figures. *P* < 0.01 and < 0.001 is indicated by two and three asterisks, respectively.

## Data Availability Statement

All datasets generated for this study are included in the article/Supplementary Material.

## Ethics Statement

The animal study was reviewed and approved by MD Anderson's Institutional Animal Care and Use Committee.

## Author Contributions

RA, HS, JP-M, MI, SK, KS, J-iA, and N-TL: performed experiments and analyzed data. KF, KS, J-iA, and N-TL: conceived and designed the experiments and wrote the manuscript. SY, NP, JB, and SL: made suggestions for the study design and experiments. JT: provided FMK-MEA and commented on the study design. All authors commented on the manuscript.

## Conflict of Interest

JT is a co-founder of Principia Biopharma, which has licensed the p90RSK inhibitor FMK-MEA. The remaining authors declare that the research was conducted in the absence of any commercial or financial relationships that could be construed as a potential conflict of interest.

## Supplementary Material

Supplementary Figure 1 p90RSK activation does not regulate LATS1 and 2 expression. **(A)** Wes analysis of p90RSK, LATS1/2, YAP, and p-YAP S127 protein expression levels in cell lysates collected from HUVECs transduced with either Ad-LacZ or Ad-p90RSK. Actin shown is a representation of the loading control present for each Wes run. **(B–E)** Quantification of protein expression levels shown in **(A)** relative to the loading control (mean ± SEM, *n* = 3). Statistical differences between two independent groups were assessed using the unpaired two-tailed Student's *t*-test. **(F)** Quantification of p-YAP S127 relative to total YAP as seen in **(A)**.

Supplementary Figure 2 Depletion of MAGI1 increases LATS1/2 expression and decreases YAP activity. **(A)** Wes analysis of MAGI1, LATS1/2, YAP, and p-YAP S127 protein expression levels in cell lysates collected from HUVECs treated with either siCTRL or siMAGI1 (100 nM, 48 h). Actin shown is a representation of the loading control present for each Wes run. **(B–E)** Quantification of protein expression levels shown in **(A)** relative to the loading control (mean ± SEM, *n* = 3). Statistical differences between two independent groups were assessed using the unpaired two-tailed Student's *t*-test. **(F)** Quantification of p-YAP S127 relative to total YAP as seen in **(A)**.

Supplementary Figure 3 Basal level TEER values from various ECIS measurments **(A)** Basal TEER values observed in HAECs transduced with Ad-MAGI1-WT, Ad-MAGI1-KR, Ad-MAGI1-S741A, Ad-p90RSK, and Ad-LacZ. **(B)** Basal TEER values observed in HAECs transduced with Ad-LacZ and Ad-DNp90RSK. **(C)** Basal TEER values observed in HAECs treated with siCTRL, siLATS1 and siLATS1/siYAP. **(D)** Basal TEER values observed in HAECs treated with siCTRL, siMAGI1, siLATS2, and siLATS2/siYAP. **(A–D)** Graphs demonstrate non-normalized resistance values (mean ± SEM). Statistical significance was assessed using ANOVA followed by Bonferroni post hoc testing for multiple group comparison. ^*^*P* < 0.05, ^**^*P* < 0.01, and ^***^*P* < 0.001.

Supplementary Figure 4 MAGI1 effects on EC permeability in acute and chronic phase after Thb stimulation.

## References

[1] Schnittler H. Contraction of endothelial cells: 40 years of research, but the debate still lives. Histochem Cell Biol. (2016) 146:651–6. 10.1007/s00418-016-1501-0 27680546

[2] Coopman P Djiane A. Adherens junction and E-cadherin complex regulation by epithelial polarity. Cell Mol Life Sci. (2016) 73:3535–53. 10.1007/s00018-016-2260-8 27151512 PMC11108514

[3] Adam AP. Regulation of endothelial adherens junctions by tyrosine phosphorylation. Mediators Inflamm. (2015) 2015:272858. 10.1155/2015/272858 26556953 PMC4628659

[4] Gavard J. Endothelial permeability and VE-cadherin: a wacky comradeship. Cell Adh Migr. (2013) 7:455–61. 10.4161/cam.27330 24430214 PMC3916348

[5] Huveneers S de Rooij J. Mechanosensitive systems at the cadherin-F-actin interface. J Cell Sci. (2013) 126:403–13. 10.1242/jcs.109447 23524998

[6] Kourtidis A Ngok SP Anastasiadis PZ. p120 catenin: an essential regulator of cadherin stability, adhesion-induced signaling, and cancer progression. Prog Mol Biol Transl Sci. (2013) 116:409–32. 10.1016/B978-0-12-394311-8.00018-2 23481205 PMC4960658

[7] Lakshmikanthan S Sobczak M Li Calzi S Shaw L Grant MB Chrzanowska-Wodnicka M. Rap1B promotes VEGF-induced endothelial permeability and is required for dynamic regulation of the endothelial barrier. J Cell Sci. (2018) 131:207605. 10.1242/jcs.207605 29222111 PMC5818062

[8] Kooistra MR Dube N Bos JL. Rap1: a key regulator in cell-cell junction formation. J Cell Sci. (2007) 120:17–22. 10.1242/jcs.03306 17182900

[9] Chrzanowska-Wodnicka M. Regulation of angiogenesis by a small GTPase Rap1. Vascul Pharmacol. (2010) 53:1–10. 10.1016/j.vph.2010.03.003 20302970

[10] Curry FR Adamson RH. Tonic regulation of vascular permeability. Acta Physiol. (2013) 207:628–49. 10.1111/apha.12076 23374222 PMC4054936

[11] Dejana E Tournier-Lasserve E Weinstein BM. The control of vascular integrity by endothelial cell junctions: molecular basis and pathological implications. Dev Cell. (2009) 16:209–21. 10.1016/j.devcel.2009.01.004 19217423

[12] Spindler V Schlegel N Waschke J. Role of GTPases in control of microvascular permeability. Cardiovasc Res. (2010) 87:243–53. 10.1093/cvr/cvq086 20299335

[13] Sakurai A Fukuhara S Yamagishi A Sako K Kamioka Y Masuda M. MAGI-1 is required for Rap1 activation upon cell-cell contact and for enhancement of vascular endothelial cadherin-mediated cell adhesion. Mol Biol Cell. (2006) 17:966–76. 10.1091/mbc.e05-07-0647 16339077 PMC1356604

[14] Feng X Jia S Martin TA Jiang WG. Regulation and involvement in cancer and pathological conditions of MAGI1, a tight junction protein. Anticancer Res. (2014) 34:3251–6. 24982328

[15] Dobrosotskaya I Guy RK James GL. MAGI-1, a membrane-associated guanylate kinase with a unique arrangement of protein-protein interaction domains. J Biol Chem. (1997) 272:31589–97. 10.1074/jbc.272.50.31589 9395497

[16] Wegmann F Ebnet K Du Pasquier L Vestweber D Butz S. Endothelial adhesion molecule ESAM binds directly to the multidomain adaptor MAGI-1 and recruits it to cell contacts. Exp Cell Res. (2004) 300:121–33. 10.1016/j.yexcr.2004.07.010 15383320

[17] Hirabayashi S Tajima M Yao I Nishimura W Mori H Hata Y. JAM4, a junctional cell adhesion molecule interacting with a tight junction protein, MAGI-1. Mol Cell Biol. (2003) 23:4267–82. 10.1128/MCB.23.12.4267-4282.2003 12773569 PMC156145

[18] Chastre E Abdessamad M Kruglov A Bruyneel E Bracke M Di Gioia Y. TRIP6, a novel molecular partner of the MAGI-1 scaffolding molecule, promotes invasiveness. FASEB J. (2009) 23:916–28. 10.1096/fj.08-106344 19017743

[19] Dobrosotskaya IY. Identification of mNET1 as a candidate ligand for the first PDZ domain of MAGI-1. Biochem Biophys Res Commun. (2001) 283:969–75. 10.1006/bbrc.2001.4880 11350080

[20] Ingham RJ Colwill K Howard C Dettwiler S Lim CS Yu J. WW domains provide a platform for the assembly of multiprotein networks. Mol Cell Biol. (2005) 25:7092–106. 10.1128/MCB.25.16.7092-7106.2005 16055720 PMC1190255

[21] Zhao B Wei X Li W Udan RS Yang Q Kim J. Inactivation of YAP oncoprotein by the Hippo pathway is involved in cell contact inhibition and tissue growth control. Genes Dev. (2007) 21:2747–61. 10.1101/gad.1602907 17974916 PMC2045129

[22] Salah Z Aqeilan RI. WW domain interactions regulate the Hippo tumor suppressor pathway. Cell Death Dis. (2011) 2:e172. 10.1038/cddis.2011.53 21677687 PMC3168995

[23] Avruch J Zhou D Fitamant J Bardeesy N Mou F Barrufet LR. Protein kinases of the Hippo pathway: regulation and substrates. Semin Cell Dev Biol. (2012) 23:770–84. 10.1016/j.semcdb.2012.07.002 22898666 PMC3489012

[24] Yu FX Guan KL. The Hippo pathway: regulators and regulations. Genes Dev. (2013) 27:355–71. 10.1101/gad.210773.112 23431053 PMC3589553

[25] Couzens AL Knight JD Kean MJ Teo G Weiss A Dunham WH. Protein interaction network of the mammalian Hippo pathway reveals mechanisms of kinase-phosphatase interactions. Sci Signal. (2013) 6:rs15. 10.1126/scisignal.2004712 24255178

[26] Wang KC Yeh YT Nguyen P Limqueco E Lopez J Thorossian S. Flow-dependent YAP/TAZ activities regulate endothelial phenotypes and atherosclerosis. Proc Natl Acad Sci USA. (2016) 113:11525–30. 10.1073/pnas.1613121113 27671657 PMC5068257

[27] Neto F Klaus-Bergmann A Ong YT Alt S Vion AC Szymborska A. YAP and TAZ regulate adherens junction dynamics and endothelial cell distribution during vascular development. Elife. (2018) 7:31037. 10.7554/eLife.31037 PMC5814147 29400648

[28] Benn A Bredow C Casanova I Vukicevic S Knaus P. VE-cadherin facilitates BMP-induced endothelial cell permeability and signaling. J Cell Sci. (2016) 129:206–18. 10.1242/jcs.179960 26598555 PMC4732303

[29] Abe JI Ko KA Kotla S Wang Y Paez-Mayorga J Shin IJ. MAGI1 as a link between endothelial activation and ER stress drives atherosclerosis. JCI Insight. (2019) 4:125570. 10.1172/jci.insight.125570 30944250 PMC6483653

[30] Carmeliet P Jain RK. Principles and mechanisms of vessel normalization for cancer and other angiogenic diseases. Nat Rev Drug Discov. (2011) 10:417–27. 10.1038/nrd3455 21629292

[31] Claesson-Welsh L. Vascular permeability-the essentials. Upsala J Med Sci. (2015) 120:135–43. 10.3109/03009734.2015.1064501 26220421 PMC4526869

[32] Martin JD Fukumura D Duda DG Boucher Y Jain RK. Reengineering the tumor microenvironment to alleviate hypoxia and overcome cancer heterogeneity. Cold Spring Harbor Perspectiv Med. (2016) 6:a027094. 10.1101/cshperspect.a027094 PMC5131751 27663981

[33] Srinivasan B Kolli AR Esch MB Abaci HE Shuler ML Hickman JJ. TEER measurement techniques for *in vitro* barrier model systems. J Lab Autom. (2015) 20:107–26. 10.1177/2211068214561025 25586998 PMC4652793

[34] Szulcek R Bogaard HJ van Nieuw Amerongen GP. Electric cell-substrate impedance sensing for the quantification of endothelial proliferation, barrier function, and motility. J Vis Exp. (2014) 85:51300. 10.3791/51300 24747269 PMC4159052

[35] Anasooya Shaji C Robinson BD Yeager A Beeram MR Davis ML Isbell CL. The tri-phasic role of hydrogen peroxide in blood-brain barrier endothelial cells. Sci Rep. (2019) 9:133. 10.1038/s41598-018-36769-3 30644421 PMC6333800

[36] Alaish SM Timmons J Smith A Buzza MS Murphy E Zhao A. Candidate genes for limiting cholestatic intestinal injury identified by gene expression profiling. Physiol Rep. (2013) 1:73. 10.1002/phy2.73 24179676 PMC3808870

[37] Cohen MS Zhang C Shokat KM Taunton J. Structural bioinformatics-based design of selective, irreversible kinase inhibitors. Science. (2005) 308:1318–21. 10.1126/science1108367 15919995 PMC3641834

[38] Kotla S Vu HT Ko KA Wang Y Imanishi M Heo KS. Endothelial senescence is induced by phosphorylation and nuclear export of telomeric repeat binding factor 2-interacting protein (TERF2IP). JCI Insight. (2019) 4:e124867. 10.1172/jci.insight.124867 31045573 PMC6538340

[39] Gou H Liang JQ Zhang L Chen H Zhang Y Li R. TTPAL promotes colorectal tumorigenesis by stabilizing TRIP6 to activate Wnt/beta-catenin signaling. Cancer Res. (2019) 79:3332–46. 10.1158/0008-5472.CAN-18-2986 31018940

[40] Dutta S Mana-Capelli S Paramasivam M Dasgupta I Cirka H Billiar K. TRIP6 inhibits Hippo signaling in response to tension at adherens junctions. EMBO Rep. (2018) 19:337–50. 10.15252/embr.201744777 29222344 PMC5797958

[41] Lv Y Kim K Sheng Y Cho J Qian Z Zhao YY. YAP controls endothelial activation and vascular inflammation through TRAF6. Circ Res. (2018) 123:43–56. 10.1161/CIRCRESAHA.118.313143 29794022 PMC6014930

[42] Le NT Heo KS Takei Y Lee H Woo CH Chang E. A crucial role for p90RSK-mediated reduction of ERK5 transcriptional activity in endothelial dysfunction and atherosclerosis. Circulation. (2013) 127:486–99. 10.1161/CIRCULATIONAHA.112.116988 23243209 PMC3574639

[43] Murakami J Li TS Ueda K Tanaka T Hamano K. Inhibition of accelerated tumor growth by blocking the recruitment of mobilized endothelial progenitor cells after chemotherapy. Int J Cancer. (2009) 124:1685–92. 10.1002/ijc.24085 19089911

[44] Chen L Lu Y Wu JM Xu B Zhang LJ Gao M. Ligustrazine inhibits B16F10 melanoma metastasis and suppresses angiogenesis induced by Vascular Endothelial Growth Factor. Biochem Biophys Res Commun. (2009) 386:374–9. 10.1016/j.bbrc.2009.06.042 19523924

[45] Tardio JC. CD34-reactive tumors of the skin. An updated review of an ever-growing list of lesions. J Cutan Pathol. (2009) 36:89–102. 10.1111/j.1600-0560.2008.01212.x 19125742

[46] Maltby S Freeman S Gold MJ Baker JH Minchinton AI Gold MR. Opposing roles for CD34 in B16 melanoma tumor growth alter early stage vasculature and late stage immune cell infiltration. PLoS ONE. (2011) 6:e18160. 10.1371/journal.pone.0018160 21494591 PMC3073928

[47] Lampugnani MG Dejana E. Interendothelial junctions: structure, signalling and functional roles. Curr Opin Cell Biol. (1997) 9:674–82. 10.1016/S0955-067480121-4 9330871

[48] Bazzoni G Dejana E. Endothelial cell-to-cell junctions: molecular organization and role in vascular homeostasis. Physiol Rev. (2004) 84:869–901. 10.1152/physrev.00035.2003 15269339

[49] Mino A Ohtsuka T Inoue E Takai Y. Membrane-associated guanylate kinase with inverted orientation (MAGI)-1/brain angiogenesis inhibitor 1-associated protein (BAP1) as a scaffolding molecule for Rap small G protein GDP/GTP exchange protein at tight junctions. Genes Cells. (2000) 5:1009–16. 10.1046/j.1365-2443.2000.00385.x 11168587

[50] Furth N Aylon Y. The LATS1 and LATS2 tumor suppressors: beyond the Hippo pathway. Cell Death Differ. (2017) 24:1488–501. 10.1038/cdd.2017.99 28644436 PMC5563998

[51] Aylon Y Michael D Shmueli A Yabuta N Nojima H Oren M. A positive feedback loop between the p53 and Lats2 tumor suppressors prevents tetraploidization. Genes Dev. (2006) 20:2687–700. 10.1101/gad.1447006 17015431 PMC1578695

[52] Li W Wang L Katoh H Liu R Zheng P Liu Y. Identification of a tumor suppressor relay between the FOXP3 and the Hippo pathways in breast and prostate cancers. Cancer Res. (2011) 71:2162–71. 10.1158/0008-5472.CAN-10-3268 21278236 PMC3070402

[53] Park GS Oh H Kim M Kim T Johnson RL Irvine KD. An evolutionarily conserved negative feedback mechanism in the Hippo pathway reflects functional difference between LATS1 and LATS2. Oncotarget. (2016) 7:24063–75. 10.18632/oncotarget.8211 27006470 PMC5029684

[54] Chen Q Zhang N Xie R Wang W Cai J Choi KS. Homeostatic control of Hippo signaling activity revealed by an endogenous activating mutation in YAP. Genes Dev. (2015) 29:1285–97. 10.1101/gad.264234.115 26109051 PMC4495399

[55] Moroishi T Park HW Qin B Chen Q Meng Z Plouffe SW. A YAP/TAZ-induced feedback mechanism regulates Hippo pathway homeostasis. Genes Dev. (2015) 29:1271–84. 10.1101/gad.262816.115 26109050 PMC4495398

[56] Siam R Harada R Cadieux C Battat R Vadnais C Nepveu A. Transcriptional activation of the Lats1 tumor suppressor gene in tumors of CUX1 transgenic mice. Mol Cancer. (2009) 8:60. 10.1186/1476-4598-8-60 19656388 PMC2731069

[57] Zhang Y Hu CF Chen J Yan LX Zeng YX Shao JY. LATS2 is de-methylated and overexpressed in nasopharyngeal carcinoma and predicts poor prognosis. BMC Cancer. (2010) 10:538. 10.1186/1471-2407-10-538 20932276 PMC2958949

[58] Zhang K Rodriguez-Aznar E Yabuta N Owen RJ Mingot JM Nojima H. Lats2 kinase potentiates Snail1 activity by promoting nuclear retention upon phosphorylation. EMBO J. (2012) 31:29–43. 10.1038/emboj.2011.357 21952048 PMC3252572

[59] Furth N Bossel Ben-Moshe N Pozniak Y Porat Z Geiger T Domany E. Down-regulation of LATS kinases alters p53 to promote cell migration. Genes Dev. (2015) 29:2325–30. 10.1101/gad.268185.115 26588988 PMC4691886

[60] Gerritsen ME Tomlinson JE Zlot C Ziman M Hwang S. Using gene expression profiling to identify the molecular basis of the synergistic actions of hepatocyte growth factor and vascular endothelial growth factor in human endothelial cells. Br J Pharmacol. (2003) 140:595–610. 10.1038/sj.bjp.0705494 14504135 PMC1574080

[61] Fukuhara S Sakurai A Sano H Yamagishi A Somekawa S Takakura N. Cyclic AMP potentiates vascular endothelial cadherin-mediated cell-cell contact to enhance endothelial barrier function through an Epac-Rap1 signaling pathway. Mol Cell Biol. (2005) 25:136–46. 10.1128/MCB.25.1.136-146.2005 15601837 PMC538793

[62] Zakharevich M Kattan JM Chen JL Lin BR Cervantes AE Chung DD. Elucidating the molecular basis of PPCD: effects of decreased ZEB1 expression on corneal endothelial cell function. Mol Vis. (2017) 23:740–52. 29046608 PMC5644665

[63] Opp D Wafula B Lim J Huang E Lo JC Lo CM. Use of electric cell-substrate impedance sensing to assess *in vitro* cytotoxicity. Biosens Bioelectron. (2009) 24:2625–9. 10.1016/j.bios.2009.01.015 19230649 PMC2668605

[64] Arndt S Seebach J Psathaki K Galla HJ Wegener J. Bioelectrical impedance assay to monitor changes in cell shape during apoptosis. Biosens Bioelectron. (2004) 19:583–94. 10.1016/S0956-566300269-0 14683642

[65] Takahashi M Berk BC. Mitogen-activated protein kinase (ERK1/2) activation by shear stress and adhesion in endothelial cells. Essential role for a herbimycin-sensitive kinase. J Clin Invest. (1996) 98:2623–31. 10.1172/JCI119083 8958227 PMC507722

[66] Baradaran-Heravi A Niesser J Balgi AD Choi K Zimmerman C South AP. Gentamicin B1 is a minor gentamicin component with major non-sense mutation suppression activity. Proc Natl Acad Sci USA. (2017) 114:3479–84. 10.1073/pnas.1620982114 28289221 PMC5380045

