# Corrigendum: p90RSK-MAGI1 Module Controls Endothelial Permeability by Post-translational Modifications of MAGI1 and Hippo Pathway

**DOI:** 10.3389/fcvm.2021.663486

**Published:** 2021-02-19

**Authors:** Rei J. Abe, Hannah Savage, Masaki Imanishi, Priyanka Banerjee, Sivareddy Kotla, Jesus Paez-Mayorga, Jack Taunton, Keigi Fujiwara, Jong Hak Won, Syed Wamique Yusuf, Nicolas L. Palaskas, Jose Banchs, Steven H. Lin, Keri L. Schadler, Jun-ichi Abe, Nhat-Tu Le

**Affiliations:** ^1^Department of Cardiovascular Sciences, Center for Cardiovascular Regeneration, Houston Methodist Research Institute, Houston, TX, United States; ^2^Department of Pediatric Research, The University of Texas MD Anderson Cancer Center, Houston, TX, United States; ^3^Department of Cardiology, The University of Texas MD Anderson Cancer Center, Houston, TX, United States; ^4^Department of Cellular and Molecular Pharmacology, University of California, San Francisco, San Francisco, CA, United States; ^5^Department of Radiation Oncology, The University of Texas MD Anderson Cancer Center, Houston, TX, United States

**Keywords:** p90RSK, SUMOylation, Hippo pathway, EC permeability, MAGI1

In the original article, we neglected to include the funder: Cancer Prevention Research Institute of Texas Training Grant (CPRIT RP170067) to Hannah Savage.

The authors apologize for this error and state that this does not change the scientific conclusions of the article in any way. The original article has been updated.

