# Supplementary figures and images for: p90RSK-MAGI1 Module Controls Endothelial Permeability by Post-translational Modifications of MAGI1 and Hippo Pathway
Source: Front Cardiovasc Med. 2020 Nov 13;7:542485. doi: 10.3389/fcvm.2020.542485 (PMC7693647; doi:10.3389/fcvm.2020.542485)

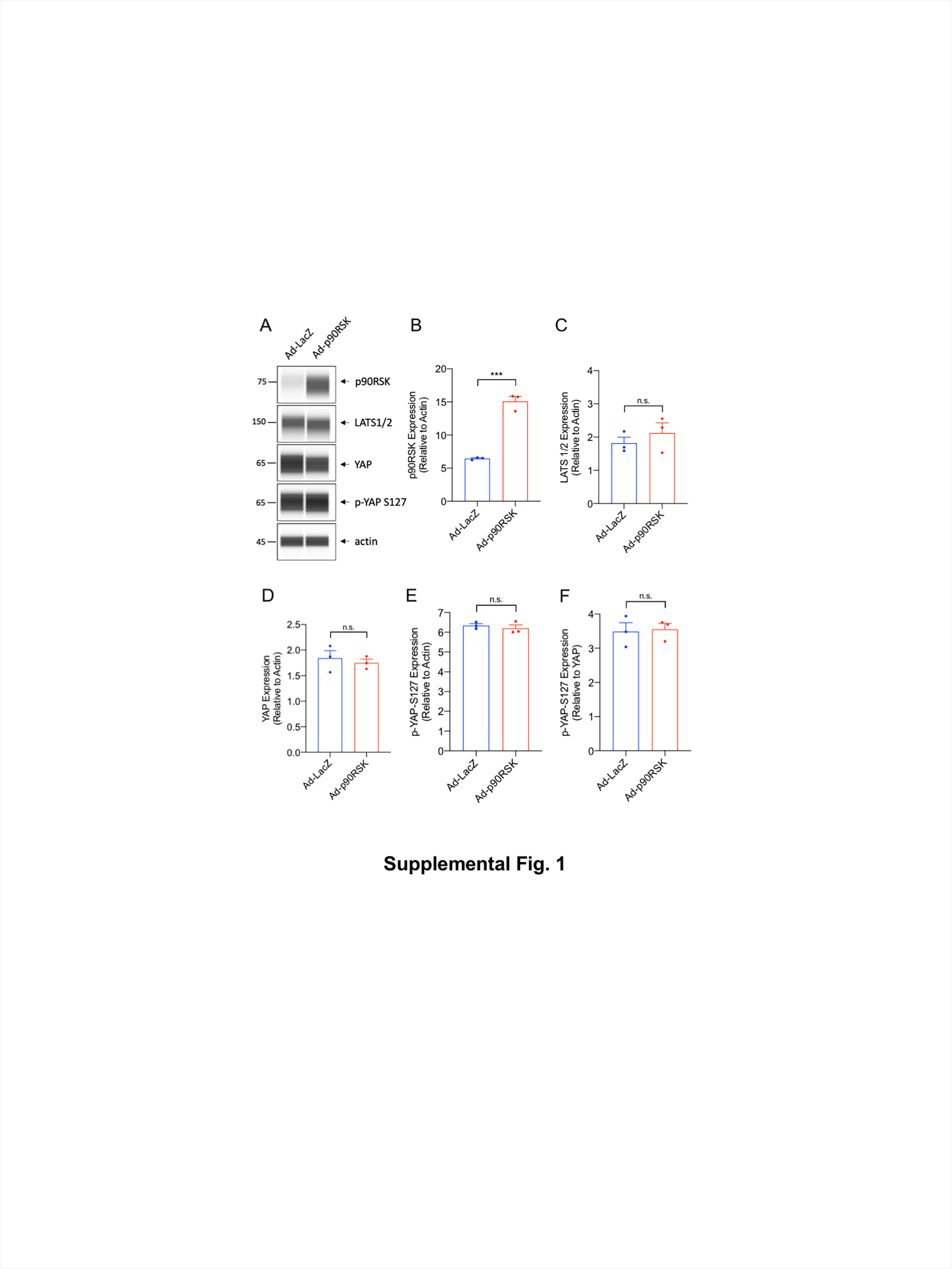

Supplement: Supplementary Figure 1 — p90RSK activation does not regulate LATS1 and 2 expression. (A) Wes analysis of p90RSK, LATS1/2, YAP, and p-YAP S127 protein expression levels in cell lysates collected from HUVECs transduced with either Ad-LacZ or Ad-p90RSK. Actin shown is a representation of the loading control present for each Wes run. (B–E) Quantification of protein expression levels shown in (A) relative to the loading control (mean ± SEM, n = 3). Statistical differences between two independent groups were assessed using the unpaired two-tailed Student's t-test. (F) Quantification of p-YAP S127 relative to total YAP as seen in (A). [file Image_1.TIF]

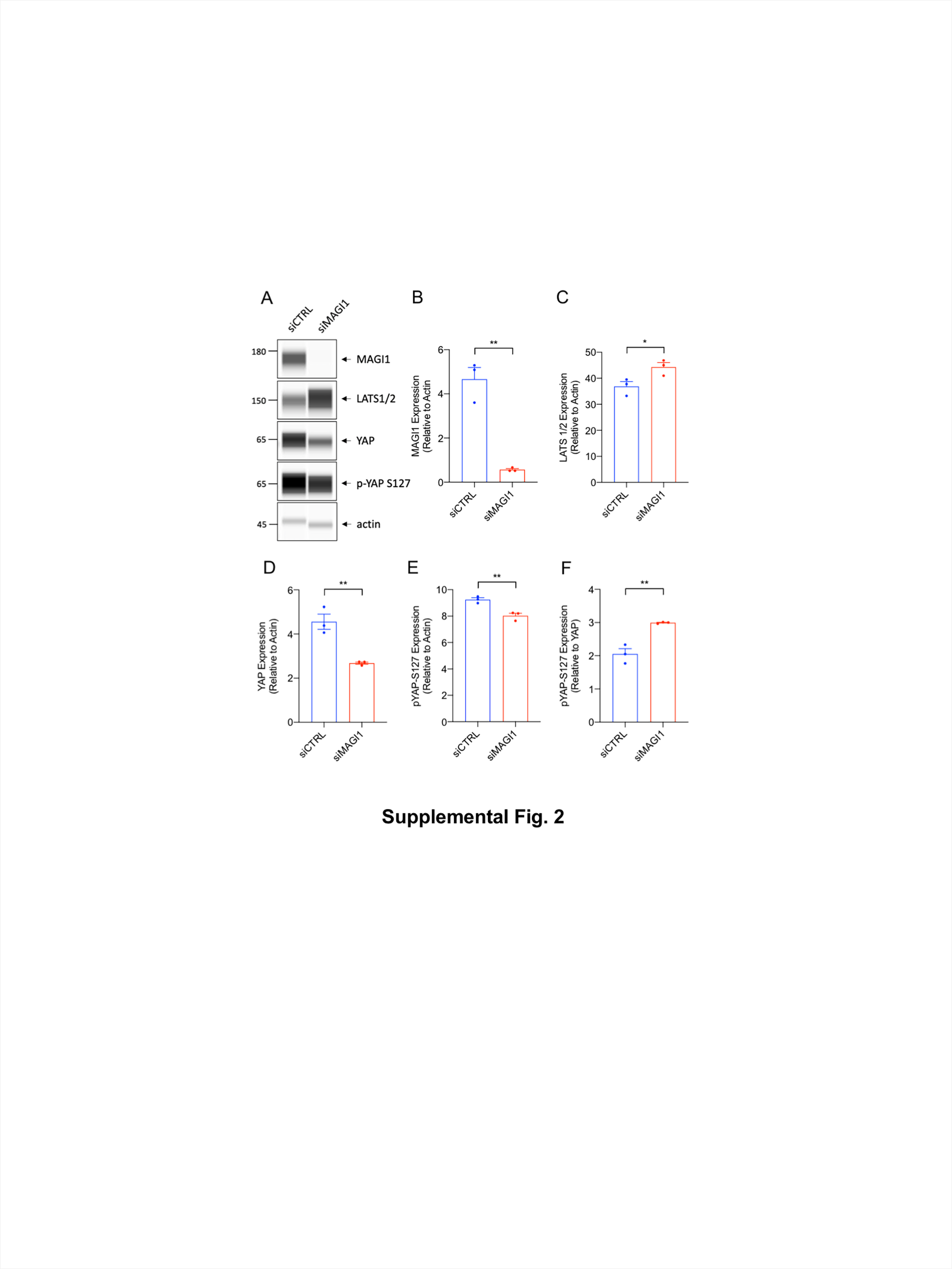

Supplement: Supplementary Figure 2 — Depletion of MAGI1 increases LATS1/2 expression and decreases YAP activity. (A) Wes analysis of MAGI1, LATS1/2, YAP, and p-YAP S127 protein expression levels in cell lysates collected from HUVECs treated with either siCTRL or siMAGI1 (100 nM, 48 h). Actin shown is a representation of the loading control present for each Wes run. (B–E) Quantification of protein expression levels shown in (A) relative to the loading control (mean ± SEM, n = 3). Statistical differences between two independent groups were assessed using the unpaired two-tailed Student's t-test. (F) Quantification of p-YAP S127 relative to total YAP as seen in (A). [file Image_2.TIF]

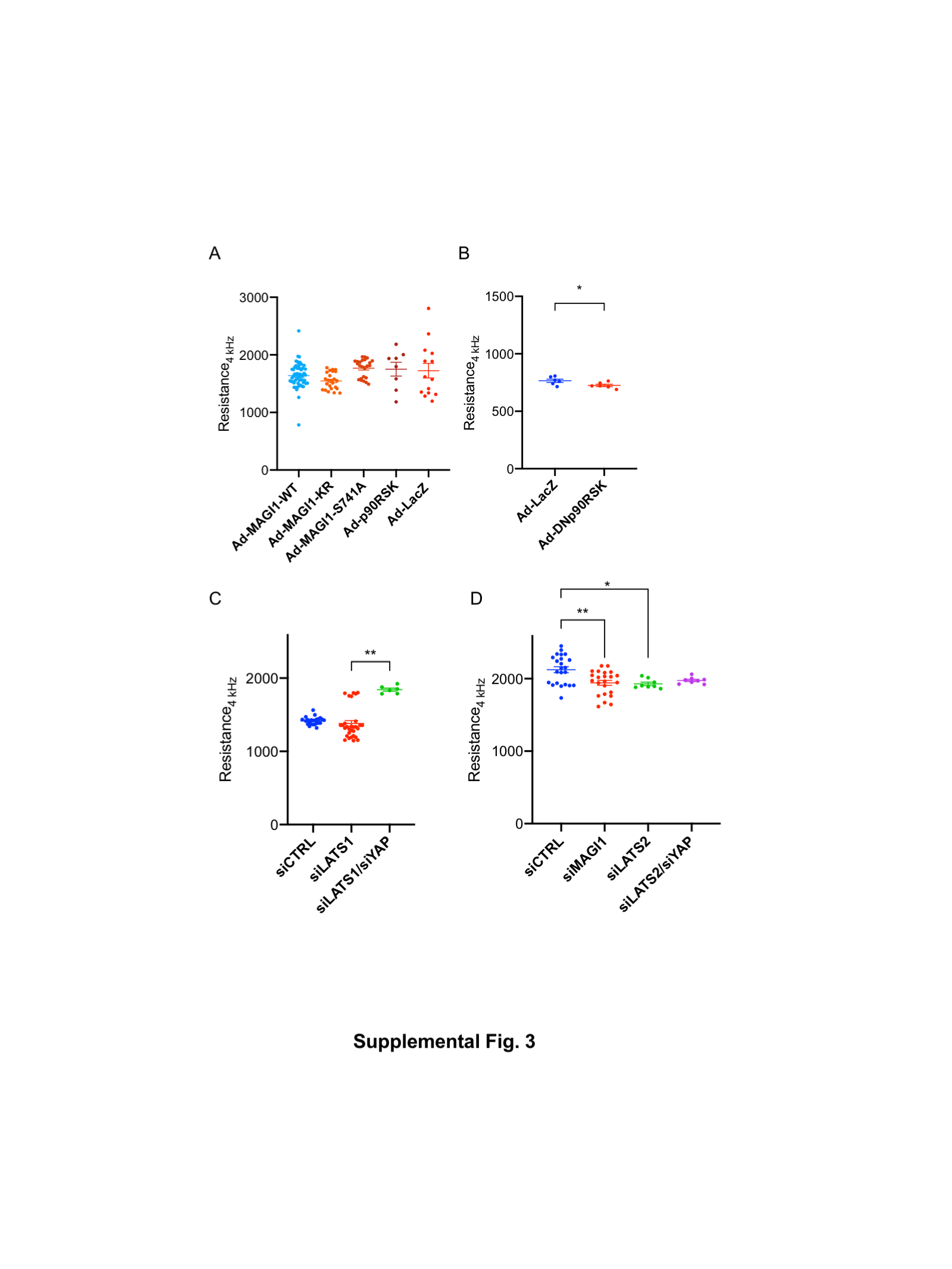

Supplement: Supplementary Figure 3 — Basal level TEER values from various ECIS measurments (A) Basal TEER values observed in HAECs transduced with Ad-MAGI1-WT, Ad-MAGI1-KR, Ad-MAGI1-S741A, Ad-p90RSK, and Ad-LacZ. (B) Basal TEER values observed in HAECs transduced with Ad-LacZ and Ad-DNp90RSK. (C) Basal TEER values observed in HAECs treated with siCTRL, siLATS1 and siLATS1/siYAP. (D) Basal TEER values observed in HAECs treated with siCTRL, siMAGI1, siLATS2, and siLATS2/siYAP. (A–D) Graphs demonstrate non-normalized resistance values (mean ± SEM). Statistical significance was assessed using ANOVA followed by Bonferroni post hoc testing for multiple group comparison. *P < 0.05, **P < 0.01, and ***P < 0.001. [file Image_3.TIFF]

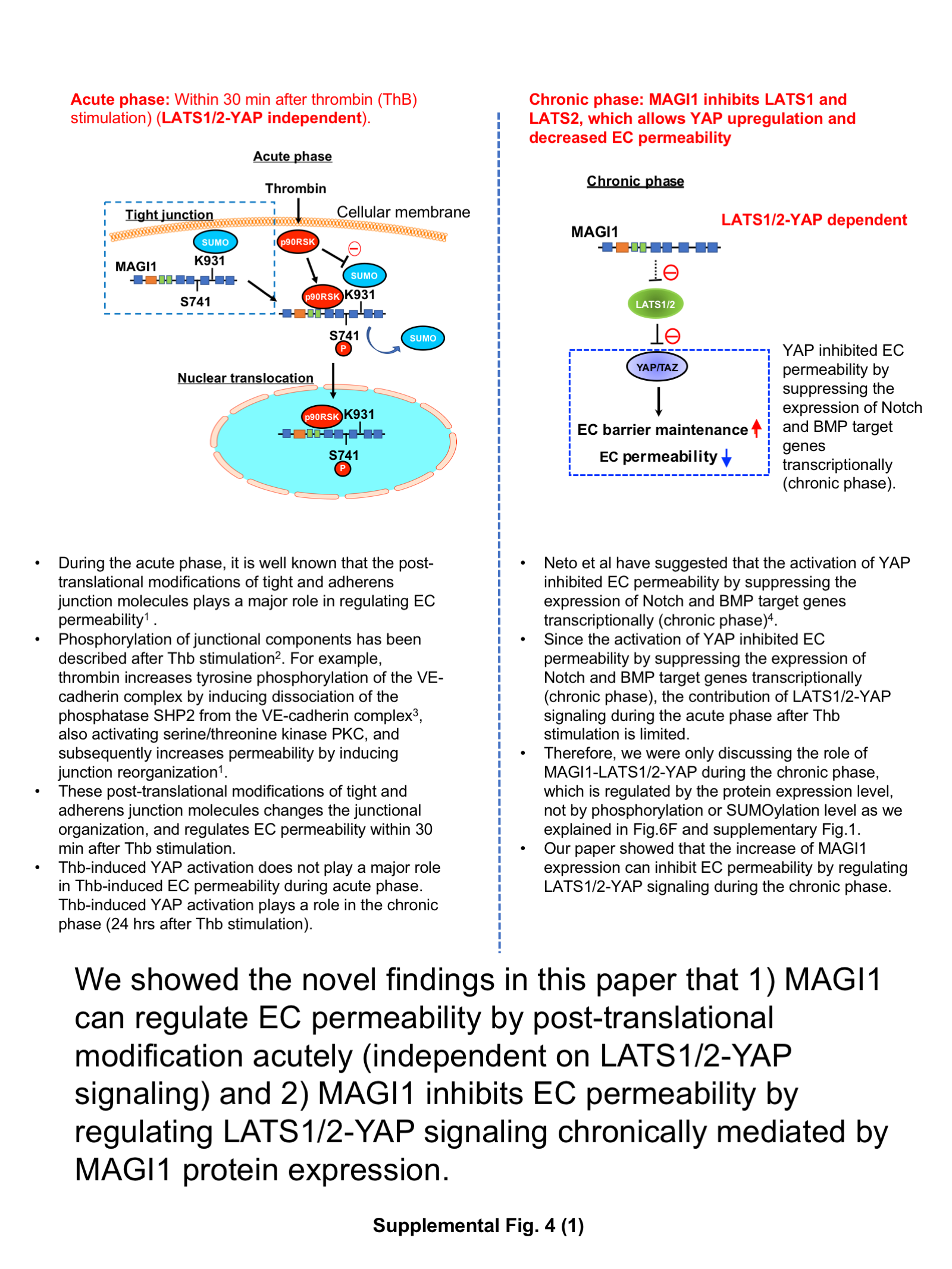

Supplement: Supplementary Figure 4 — MAGI1 effects on EC permeability in acute and chronic phase after Thb stimulation. [file Image_4.TIFF]

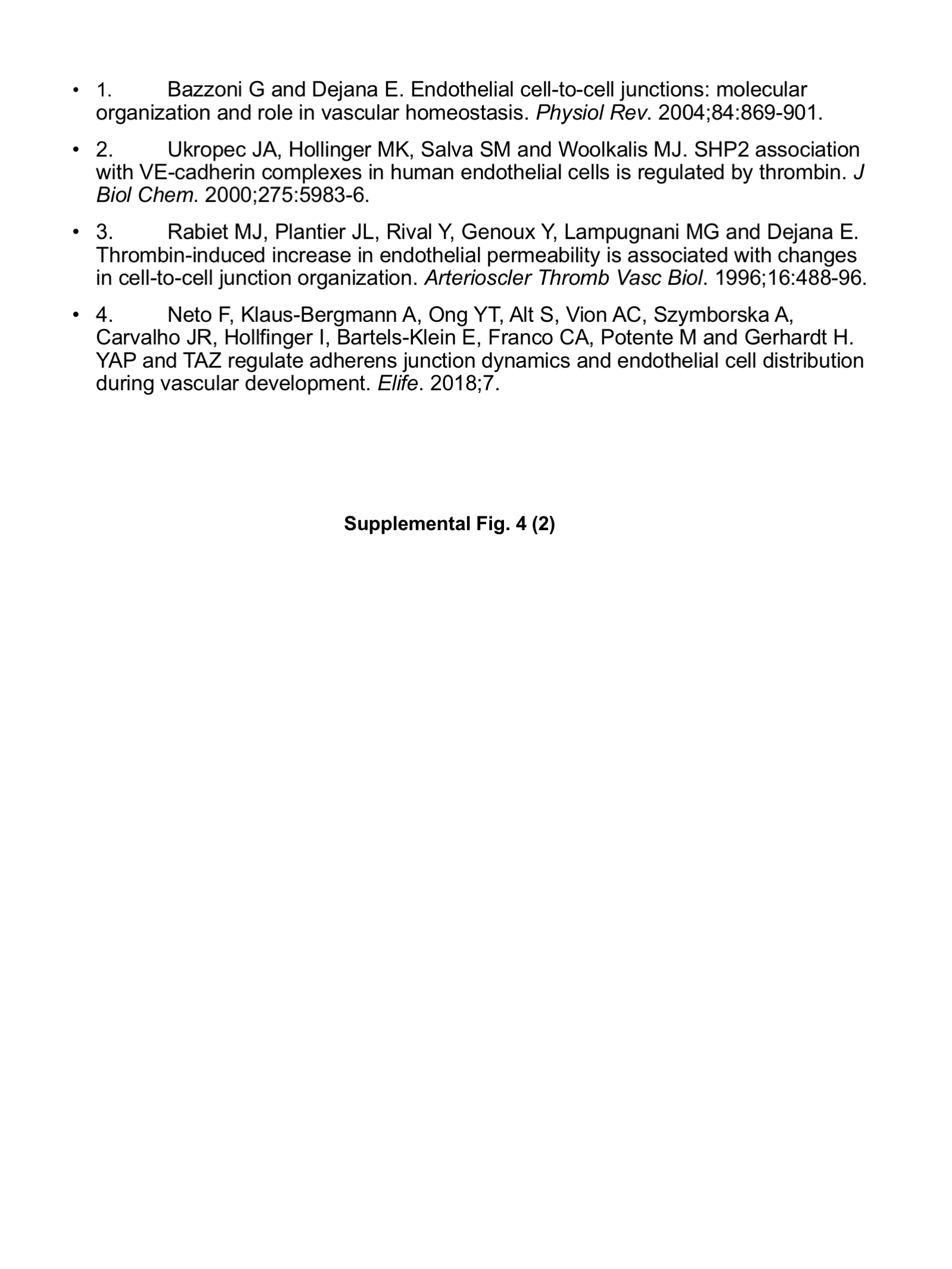

Supplement: Supplementary file 5 [file Image_5.TIFF]
